# Long Non‐Coding RNA *IGFRIL* Couples with PTBP1 to Destabilize *IGFBP3* mRNA to Promote the IGF1R‐AKT‐mTOR Axis and Hepatocellular Carcinoma

**DOI:** 10.1002/advs.202507676

**Published:** 2025-07-21

**Authors:** Jing Zhang, Chengming Gao, Haibei Li, Yuying Chen, Liting Yang, Qian Jin, Lan Feng, Xia Wang, Xinyi Liu, Hongxia Chen, Rong Ye, Yuanchao Xue, Huiyun Wang, Musheng Zeng, Ming Yang, Huaiqiang Ju, Chengzhi Gao, Guangming Zhou, Qi Zhang, Qian Zhang, Geng Qin, Yuanfeng Li, Yahui Wang, Aiqing Yang, Anfeng Si, Qingfeng Song, Fuchu He, Pengbo Cao, Gangqiao Zhou

**Affiliations:** ^1^ State Key Laboratory of Medical Proteomics National Center for Protein Sciences at Beijing Beijing Institute of Radiation Medicine Beijing 100850 China; ^2^ Institute of Life Science and Green Development College of Life Sciences Hebei University Baoding City Hebei Province 071000 China; ^3^ Department of Environment and Health Military Medical Sciences Academy Tianjin 300132 China; ^4^ Hengyang Medical School University of South China Hengyang City Hunan Province 421001 China; ^5^ Department of Neurosurgery Xiangya Hospital Central South University Changsha City Hunan Province 410008 China; ^6^ Jiangsu Key Laboratory of Biological Cancer Therapy Xuzhou Medical College Xuzhou City Jiangsu Province 221006 China; ^7^ Key Laboratory of RNA Biology Institute of Biophysics Chinese Academy of Sciences Beijing 100101 China; ^8^ State Key Laboratory of Oncology in South China Collaborative Innovation Center for Cancer Medicine Sun Yat‐Sen University Cancer Center Guangzhou City Guangdong Province 510060‌ China; ^9^ Shandong Provincial Key Laboratory of Precision Oncology Shandong Cancer Hospital and Institute Jinan City Shandong Province 250117 China; ^10^ Department of Biomed Medical College of Guizhou University Guiyang City Guizhou Province 550025 China; ^11^ Department of Research Laboratory Management West China Hospital of Sichuan University Chengdu City Sichuan Province 610041 China; ^12^ Department of Surgical Oncolog, Affiliated Jinling Hospital Medical School of Nanjing University Nanjing City Jiangsu Province 210015 China; ^13^ Department of Invasive Technology Affiliated Cancer Hospital of Guangxi Medical University Guangxi Zhuang Autonomous Region Nanning City 530012 China; ^14^ State Key Laboratory of Medical Proteomics National Center for Protein Sciences at Beijing Beijing Institute of Lifeomics Beijing 102206 China; ^15^ Collaborative Innovation Center for Personalized Cancer Medicine Center for Global Health School of Public Health Nanjing Medical University Nanjing City Jiangsu Province 211166 China

**Keywords:** hepatocellular carcinoma (HCC), IGF1R‐AKT‐mTOR signaling, IGFBP3, long non‐coding RNA IGFRIL, PTBP1

## Abstract

The incomplete understanding of the IGFR pathway activation mechanism limits its clinical application in hepatocellular carcinoma (HCC). Here, a transcriptome‐wide screening is performed and a novel HCC‐associated lncRNA, named *IGFR‐inducing lncRNA* (*IGFRIL*) is identified. *IGFRIL* is frequently upregulated in HCC tissues and predicts poor clinical outcomes. It is revealed that *IGFRIL* plays an oncogenic role in the development of HCC. Mechanistically, *IGFRIL* serves as a scaffold to recruit PTBP1, destabilizing *IGFBP3* mRNA and thereby overactivating the IGF1R‐AKT‐mTOR signaling in HCC cells. Furthermore, it is observed that the inhibitors against IGF1R or mTOR exhibit suppressive effects on patient‐derived tumor xenograft tumors with high *IGFRIL* expression, through simultaneous blocking of the IGF1R‐AKT‐mTOR signaling pathway. In summary, this study identifies *IGFRIL* as a novel non‐coding activator of the IGF1R pathway, providing a promising new therapeutic target for HCC patients.

## Graphical abstract

The limited understanding of IGFR pathway activation hinders its clinical application in HCC. Here, *IGFRIL* is identified as a novel non‐coding activator of the IGF1R, which recruits PTBP1 to destabilize *IGFBP3* mRNA and activates IGF1R, and proposes *IGFRIL* as a novel actionable target for HCC patients.

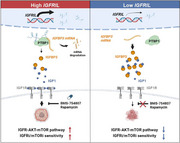

## 1 Introduction

Primary liver cancer (PLC) is the third leading cause of cancer‐related deaths globally, with over 900000 new cases and 800000 deaths annually.^[^ ^1^ ^]^ Hepatocellular carcinoma (HCC) accounts for 75% of PLC cases. Despite therapeutic advancements, high rates of recurrence and metastasis limit the 5‐year survival of HCC patients.^[^ ^2^, ^3^ ^]^ The molecular pathogenesis of HCC is highly variable, influenced by diverse genotoxic insults and etiologies. The pathophysiology and underlying drivers of HCC are not yet fully understood.

The insulin‐like growth factor (IGF) signaling pathway, comprising ligands, receptors, and six high‐affinity IGF binding proteins (IGFBP1‐6), regulates cell proliferation, differentiation, migration, and apoptosis.^[^ ^4^ ^]^ Dysregulation of this pathway is a hallmark of cancer, including HCC. While epigenetic mechanisms, such as hepatitis B virus (HBV) X protein‐mediated hypomethylation of the *IGF2* promoter,^[^ ^5^ ^]^ contribute to IGF pathway activation in HCC, the significant downregulation of IGFBP3 in ≈75% of HCC cases suggests additional mechanisms.^[^ ^6^ ^]^ While hypermethylation of the IGFBP3 promoter occurs in 33% cases,^[^ ^6^ ^]^ other factors likely contribute to its decreased expression in HCC.

Long non‐coding RNAs (lncRNAs) are non‐coding transcripts exceeding 200 nucleotides that regulate various biological processes, including cell proliferation, apoptosis, and migration.^[^ ^7^, ^8^ ^]^ LncRNAs have been implicated in numerous diseases, including HCC.^[^ ^9^, ^10^, ^11^ ^]^ While several HCC‐associated lncRNAs have been studied, the majority remain poorly understood. Additionally, while microRNAs have been shown to regulate the IGF1R signaling, the role of lncRNAs in this process remains largely unexplored in HCC.

Here, we identified a novel HCC‐associated lncRNA, *IGFR‐*inducing *lncRNA* (*IGFRIL*), which is upregulated in HCC tissues and predicts poor patient outcomes. Functionally, *IGFRIL* promotes HCC tumorigenesis by destabilizing *IGFBP3* mRNA through its interaction with PTBP1, thereby activating the IGF1R‐AKT‐mTOR signaling pathway. Importantly, HCC cells with high *IGFRIL* expression are more sensitive to IGF1R and mTOR inhibitors. Our findings suggest that *IGFRIL* may serve as a potential therapeutic target for HCC.

## 2 Results

### 2.1 Identification of *IGFRIL* as a Candidate Functional lncRNA Relevant to HCC

**Figure 1:**
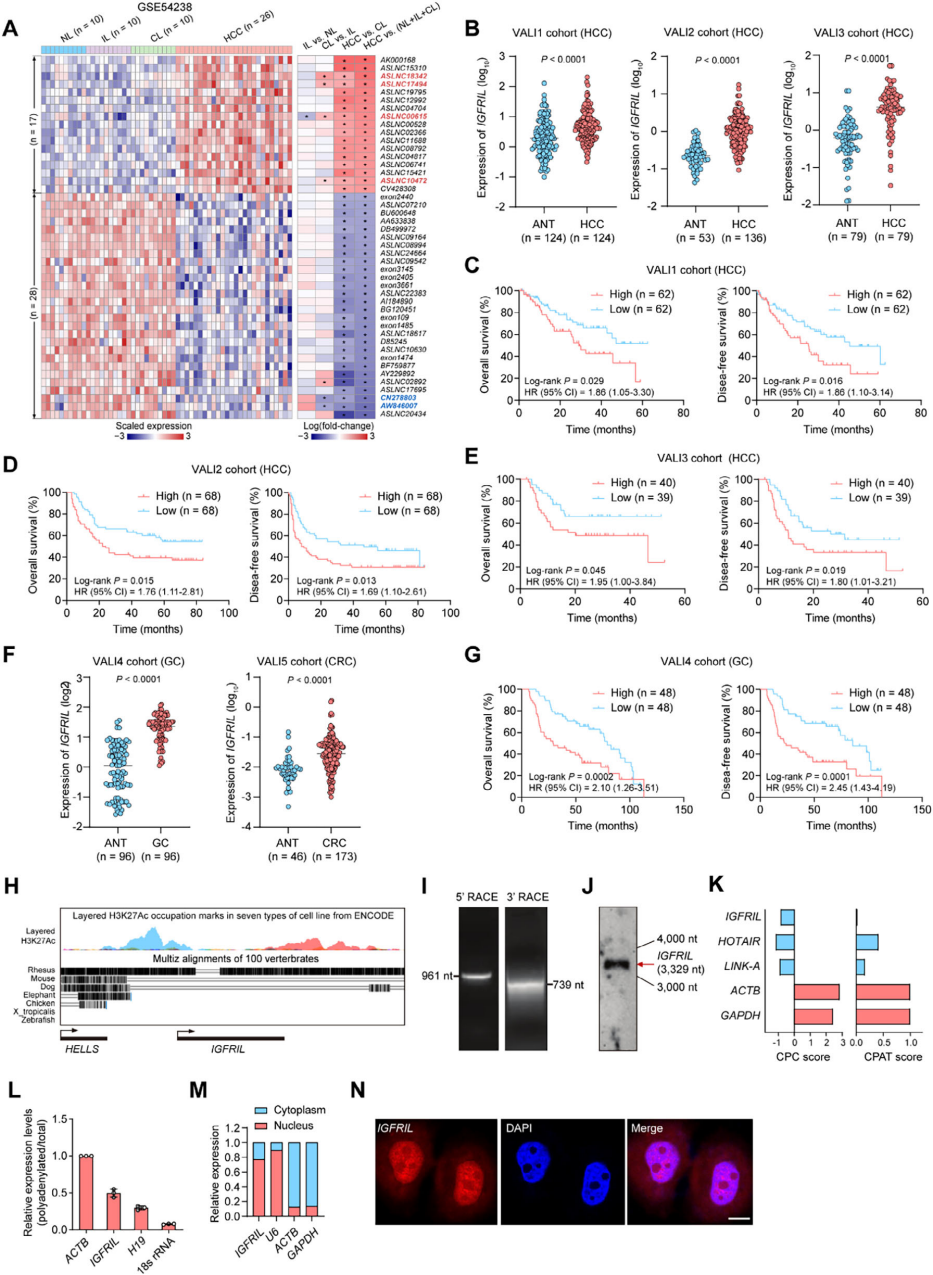
Identification of *IGFRIL* as an HCC‐associated lncRNA. A) Heatmap showing the differentially expressed lncRNAs in HCC tumors compared to non‐tumor liver tissues (normal liver [NL], inflammatory liver [IL], and cirrhotic liver [CL]; GSE54238). The dysregulated lncRNAs were ranked according to their fold‐changes of HCC versus (NL+ IL + CL). LncRNAs with a gradually increasing or decreasing expression pattern during tumor progression are highlighted in red or blue, respectively. B) *IGFRIL* expression levels in tumor and adjacent non‐tumor tissues (ANT) from three HCC validation cohorts (VALI1‐3), as determined by qRT‐PCR. C–E) Kaplan–Meier survival curves showing the overall survival (OS) and disease‐free survival (DFS) of HCC patients in the VALI1, VALI2, and VALI3 cohorts, stratified by *IGFRIL* expression levels (log‐rank test). F) *IGFRIL* expression levels in tumor and adjacent non‐tumor tissues from gastric cancer (GC) and colorectal cancer (CRC) cohorts (VALI4 and VALI5), as determined by qRT‐PCR. G) Kaplan–Meier survival curves showing the OS and DFS of GC patients in the VALI4 cohort, stratified by *IGFRIL* expression levels. H) Schematic representation of the *IGFRIL* locus, including conservation scores and H3K27Ac histone modification marks. The ENCODE project provides H3K27ac histone mark data from seven cell lines, which can be accessed through the UCSC Genome Browser (Track name: H3K27Ac Mark on 7 cell lines from ENCODE). This H3K27ac mark is located near active regulatory elements and is associated with increased transcription and active enhancers. I,J) RACE and Northern blot analyses of *IGFRIL* in HepG2 cells. K) Prediction of *IGFRIL* protein‐coding potential using CPC and CPAT. L) Relative abundance of *IGFRIL* in total and polyadenylated RNA from HepG2 cells. M,N) Subcellular localization of *IGFRIL* in HepG2 cells, as determined by qRT‐PCR analysis of nuclear and cytoplasmic fractions (M) or RNA FISH (N). Scale bar in (N), 20 µm.^*^ *P *< 0.05 by Student's *t*‐test.

We analyzed a human lncRNA microarray dataset (GSE54238)^[^ ^12^ ^]^ comprising 10 normal liver (NL), 10 chronic inflammatory liver (IL), 10 cirrhotic liver (CL), and 26 HCC tissues. By comparing HCC tissues with non‐tumor liver tissues, we identified 45 differentially expressed lncRNAs (DELs) and 158 differentially expressed protein‐coding mRNAs (DEMs) (Table S1, Supporting Information). Notably, many known HCC‐associated genes, such as *AFP*, *AKR1B10*, *GPC3*, *AURKA*, *PLK1*, *ROBO1*, *PRC2*, and *EZH2*, were identified among the DEMs. Among those DELs, the expressions of 6 ones gradually increased or decreased as the disease progressed from IL to CL and then to HCC (**Figure** **1**A). Notably, *ASLNC18342* (*IGFRIL*) ranked the highest and was therefore selected as a representative for further studies (Figure 1A). Consistent upregulation of *IGFRIL* was observed in the GSE84006 dataset (Figure S1A, Supporting Information).

We further validated *IGFRIL* overexpression in three additional HCC validation cohorts (VALI1‐3; Figure 1B, Table S2, Supporting Information). *IGFRIL* was significantly upregulated in tumor tissues of all cohorts, regardless of HBV infection status (Figure S1B, Supporting Information). Approximately 66% of HCC patients exhibited more than a twofold increase in *IGFRIL* expression in tumor tissues compared to non‐tumor tissues (Figure 1B). High *IGFRIL* expression correlated with elevated AFP levels in the VALI1 cohort and advanced TNM stage in the VALI2 cohort (Table S3, Supporting Information). Kaplan–Meier analyses revealed that patients with high *IGFRIL* expression had significantly shorter overall (OS) and disease‐free survival (DFS) in all three cohorts (Figure 1C–E). Multivariate Cox regression analysis identified high *IGFRIL* expression as an independent prognostic factor for decreased OS and DFS, particularly in the VALI1 and VALI3 cohorts (Table S4, Supporting Information).

We further assessed *IGFRIL* expression in gastric cancer (GC) and colorectal cancer (CRC) cohorts (VALI4 and VALI5, respectively; Table S2, Supporting Information). *IGFRIL* was significantly upregulated in tumor tissues of both GC and CRC patients compared to paired non‐tumor tissues (Figure 1F). Moreover, high *IGFRIL* expression predicted poor clinical outcomes in GC patients (Figure 1G). These findings suggest that *IGFRIL* may have an oncogenic role in multiple cancer types and could serve as a prognostic biomarker for HCC and other cancers.

*IGFRIL* is located on chromosome 10q23, downstream of the protein‐coding gene *HELLS* (Figure 1H). While a previous study suggested a potential overlap between the lncRNA *AK091544* (i.e., *IGFRIL*) and the 3′ UTR of a low‐abundance *HELLS* isoform in human dermal fibroblasts,^[^ ^13^ ^]^ our analysis indicates that *IGFRIL* is independently transcribed in HCC cells. However, the significant histone H3K27 acetylation signals and independent long‐read RNA‐seq reads mapped to the *IGFRIL* locus in HepG2 cells support that *IGFRIL* is independently transcribed in HCC cells (Figure 1H; Figure S1C, Supporting Information). Furthermore, no significant correlation was observed between *IGFRIL* and *HELLS* mRNA levels (GSE84006; Figure S1D, Supporting Information), and manipulating *IGFRIL* expression did not affect the expression of neighboring 8 genes (including *HELLS*, *PLCE1*, *NOC3L*, *TBC1D12*, *CYP2C18*, *CYP2C19*, *CYP2C9* and *CYP2C8*) within the 1 megabase (Mb) region centered around the *IGFRIL* locus in HepG2 and MHCC97H cells (Figure S1E,F, Supporting Information). These findings strongly suggest that *IGFRIL* is an independently transcribed lncRNA that does not exert regulatory effects on neighboring genes.

Next, using rapid amplification of cDNA ends (RACE) and Northern blotting, we confirmed that *IGFRIL* is a 3329 nucleotide (nt) transcript (Figure 1I,J; Figure S1G, Supporting Information). Computational analyses using CPC^[^ ^14^ ^]^ and CPAT^[^ ^15^ ^]^ predicted that *IGFRIL* has a low protein‐coding potential (Figure 1K). It was shown that *IGFRIL* is transcribed with a poly(A) tail (Figure 1L). Secondary structure prediction using RNAfold revealed five distinct stem‐loop structures, suggesting potential interactions with other biomolecules (Figure S1H, Supporting Information). Subcellular localization studies using nuclear/cytoplasmic fractionation and RNA fluorescence in situ hybridization (FISH) showed that *IGFRIL* is present in both the cytoplasm and nucleus of HepG2 cells (Figure 1M,N).

### 2.2 *IGFRIL* Promotes HCC Cell Growth and Metastasis

**Figure 2:**
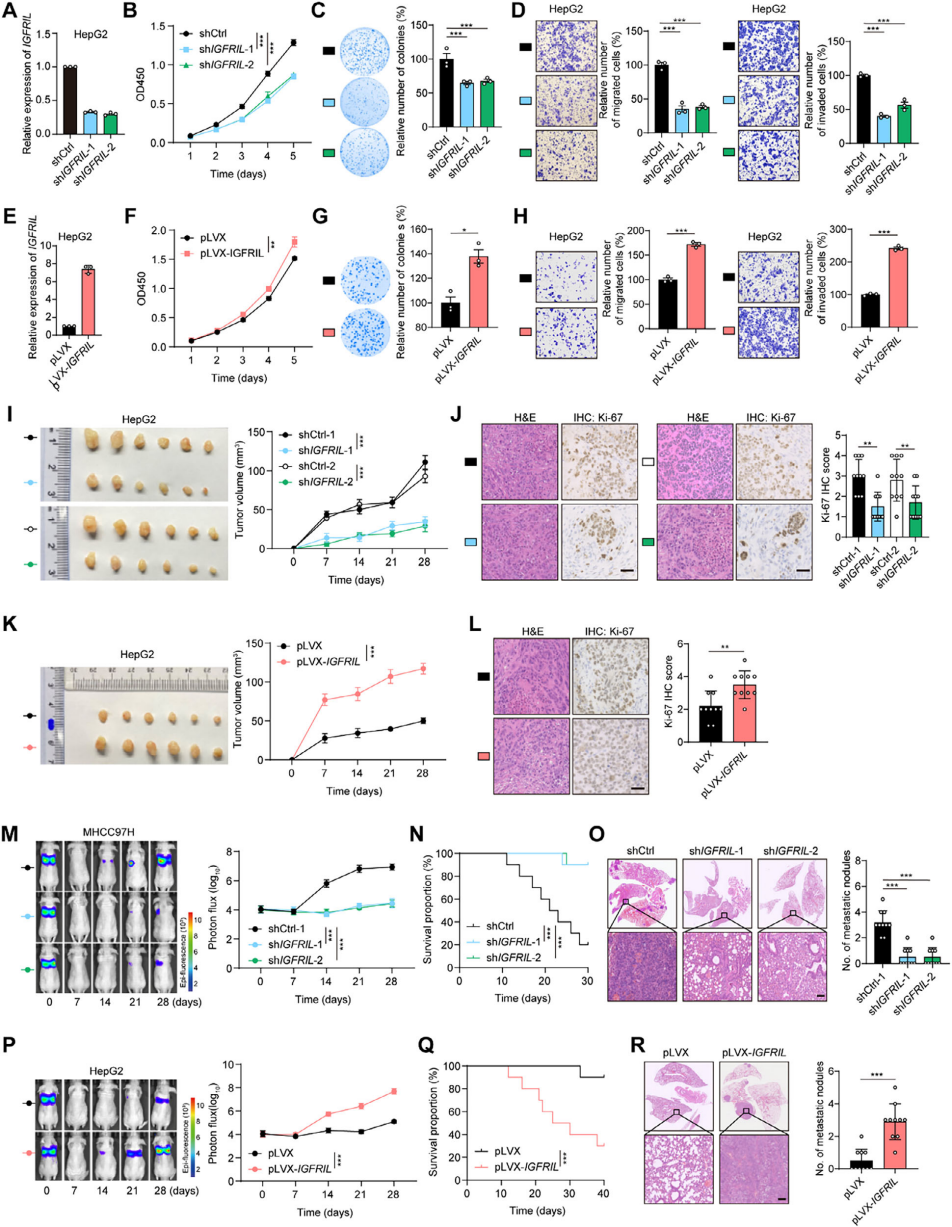
*IGFRIL* plays an oncogenic role in HCC in vitro and in vivo. A) Confirmation of the knockdown effect on the expression of *IGFRIL* by using qRT‐PCR assays. B–D) *IGFRIL* knockdown suppresses cell proliferation, colony formation, migration (24 h), and invasion (48 h) in HepG2 cells. E) Confirmation of the overexpression effect on the expression of *IGFRIL* by using qRT‐PCR assays. F–H) *IGFRIL* overexpression promotes cell proliferation, colony formation, migration, and invasion in HepG2 cells. I,J) *IGFRIL* knockdown inhibits HepG2 cell‐derived subcutaneous tumor growth and reduces Ki‐67 staining in tumor tissues (*n* = 6). K,L) *IGFRIL* overexpression promotes HepG2 cell‐derived subcutaneous tumor growth (*n* = 6). M–O) *IGFRIL* knockdown inhibits MHCC97H cell‐derived tumor metastasis and prolongs the survival of mice. Luciferase‐labeled *IGFRIL*‐knockdown or control MHCC97H cells were injected via the tail vein into the nude mice (*n* = 10). P–R) *IGFRIL* overexpression promotes HepG2 cell‐derived tumor metastasis (*n* = 10). Scale bars in (J,L,O,R), 100 µm. Data are shown as mean ± SEM, ^**^ *P *< 0.01, ^***^ *P *< 0.001 by 1‐way ANOVA (B–D,M), Student's *t‐*test (F–I,K,P), Wilcoxon signed‐rank test (J,L,O,R) or log‐rank test (N,Q).

We next assessed the tumorigenic role of *IGFRIL* in HCC cells. Knockdown of *IGFRIL* in HepG2, MHCC97H, and Huh7 cells significantly reduced cell proliferation, colony formation, migration, and invasion (**Figure** **2**A–D; Figure S2A, Supporting Information). Conversely, overexpression of *IGFRIL* in HepG2, Huh7, and JHH2 cells significantly enhanced these cellular functions (Figure 2E–H; Figure S2B, Supporting Information).

We further investigated the tumorigenic role of *IGFRIL* in vivo. Knockdown of *IGFRIL* in HepG2 cells significantly reduced tumor growth in a subcutaneous xenograft model (Figure 2I). Ki‐67 staining confirmed decreased tumor cell proliferation in *IGFRIL*‐knockdown tumors (Figure 2J; Figure S2C, Supporting Information). Conversely, overexpression of *IGFRIL* in HepG2 cells promoted tumor growth (Figure 2K,L; Figure S2D, Supporting Information). To assess the impact of *IGFRIL* on metastasis, we injected luciferase‐labeled *IGFRIL*‐knockdown MHCC97H cells into the tail vein of nude mice. Knockdown of *IGFRIL* significantly reduced lung metastasis formation, prolonged survival, and decreased the number of lung metastatic nodules (Figure 2M–O). Conversely, overexpression of *IGFRIL* in HepG2 cells increased lung metastasis and shortened survival (Figure 2P–R). Collectively, these findings demonstrate the oncogenic role of *IGFRIL* in HCC progression.

### 2.3 *IGFRIL* Physically Interacts with PTBP1 to Exert Oncogenic Roles

**Figure 3:**
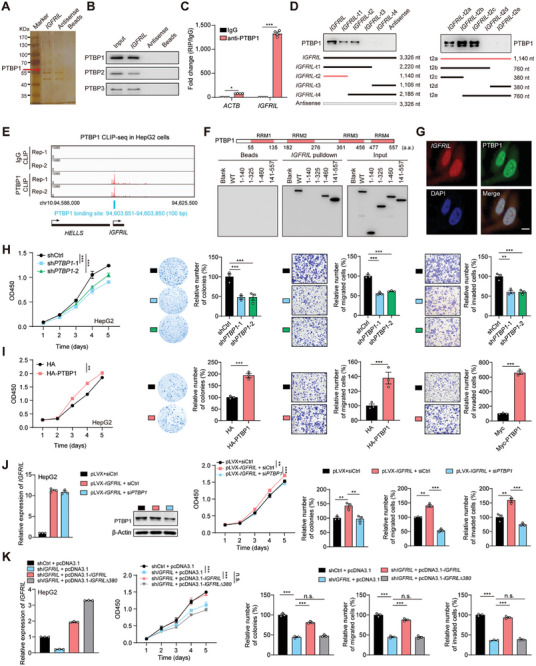
*IGFRIL* interacts with PTBP1 and relies on PTBP1 to play oncogenic roles in HCC cells. A) Silver staining of *IGFRIL*‐associated proteins after RNA pull‐down, with the identified PTBP proteins indicated. B) Immunoblotting (IB) assays of PTBP1, PTBP2 and PTBP3 pulled down by *IGFRIL*. C) RNA immunoprecipitation (RIP) assay validating the *IGFRIL*‐PTBP1 interaction. D) Mapping the PTBP1‐binding region on *IGFRIL* using truncated *IGFRIL* mutants. E) CLIP‐seq analysis showing PTBP1 binding to the 5′ end of *IGFRIL*. F) Mapping the *IGFRIL*‐binding region on PTBP1 using truncated PTBP1 mutants. WT, wild‐type. G) Colocalization of *IGFRIL* and PTBP1 in HepG2 cells, as shown by RNA FISH and immunofluorescence. Scale bar, 20 µm. H,I) The tumorigenic role of PTPB1 in HepG2 cells. J) *PTBP1* knockdown abolishes the tumor‐promoting effects of *IGFRIL* overexpression on HepG2 cells. K) Re‐introduction of full‐length *IGFRIL*, but not the *IGFRIL△380* mutant, rescues the decreased malignant phenotypes by *IGFRIL* knockdown in HepG2 cells. Data are shown as mean ± SEM, ^*^ *P *< 0.05, ^**^ *P *< 0.01, ^***^ *P *< 0.001 by 1‐way ANOVA (H,J,K) or Student's *t‐*test (C,I). n.s., not significant.

Next, we sought to elucidate the molecular mechanism underlying *IGFRIL*’s oncogenic role in HCC. RNA pull‐down coupled with mass spectrometry identified three mRNA metabolism‐associated polypyrimidine tract‐binding proteins^[^ ^16^ ^]^ (PTBP1, PTBP2, and PTBP3) as *IGFRIL*‐interacting proteins (**Figure** **3**A,B, Table S5, Supporting Information). Among these, PTBP1 was the most abundant (Figure S3A, Table S5, Supporting Information). RNA immunoprecipitation (RIP) assays confirmed the interaction between *IGFRIL* and PTBP1 (Figure 3C). Further, RNA pull‐down assays using truncated *IGFRIL* fragments revealed that the 5′ end of *IGFRIL* (containing one of the five predicted stem‐loop structures) is essential for PTBP1 binding (Figure 3D). Cross‐linking immunoprecipitation sequencing (CLIP‐seq) analyses identified abundant PTBP1 binding to the 5′ end of *IGFRIL* (Figure 3E). The PTBP1 protein contains four RNA recognition motifs (RRMs), and each one can bind to a CUCUCU oligonucleotide motif.^[^ ^17^ ^]^ Domain mapping experiments demonstrated that the RRM3 domain of PTBP1 is crucial for this interaction (Figure 3F). Subcellular localization studies using RNA FISH confirmed the colocalization of *IGFRIL* and PTBP1 in both the nucleus and cytoplasm of HepG2 cells (Figure 3G). These findings collectively indicate a physical interaction between *IGFRIL* and PTBP1.

We next explored the functional significance of this association. Neither *IGFRIL* nor PTBP1 affected the expression levels of the other in HepG2, MHCC97H, and Huh7 cells (Figure S3B–E, Supporting Information). Knockdown of *PTBP1* significantly reduced cell proliferation, colony formation, migration, and invasion in HepG2 and MHCC97H cells (Figure 3H; Figure S3F, Supporting Information). Conversely, PTBP1 overexpression promoted these cellular functions in HepG2 and Huh7 cells (Figure 3I; Figure S3G, Supporting Information), phenocopying the oncogenic role of *IGFRIL* in HCC. Furthermore, *PTBP1* knockdown attenuated the enhanced cell proliferation, colony formation, migration, and invasion induced by *IGFRIL* overexpression (Figure 3J; Figure S4A,B, Supporting Information). Meanwhile, overexpression of wild‐type *IGFRIL*, but not a mutant lacking the PTBP1‐binding region (*IGFRIL*△380), rescued the inhibitory effects of *IGFRIL* knockdown on cell growth, migration, and invasion (Figure 3K; Figure S4C,D, Supporting Information). These results collectively indicate that *IGFRIL* interacts with PTBP1 to exert its oncogenic roles.

### 2.4 *IGFRIL* Couples with PTBP1 to Induce the AKT‐mTOR Signaling

**Figure 4:**
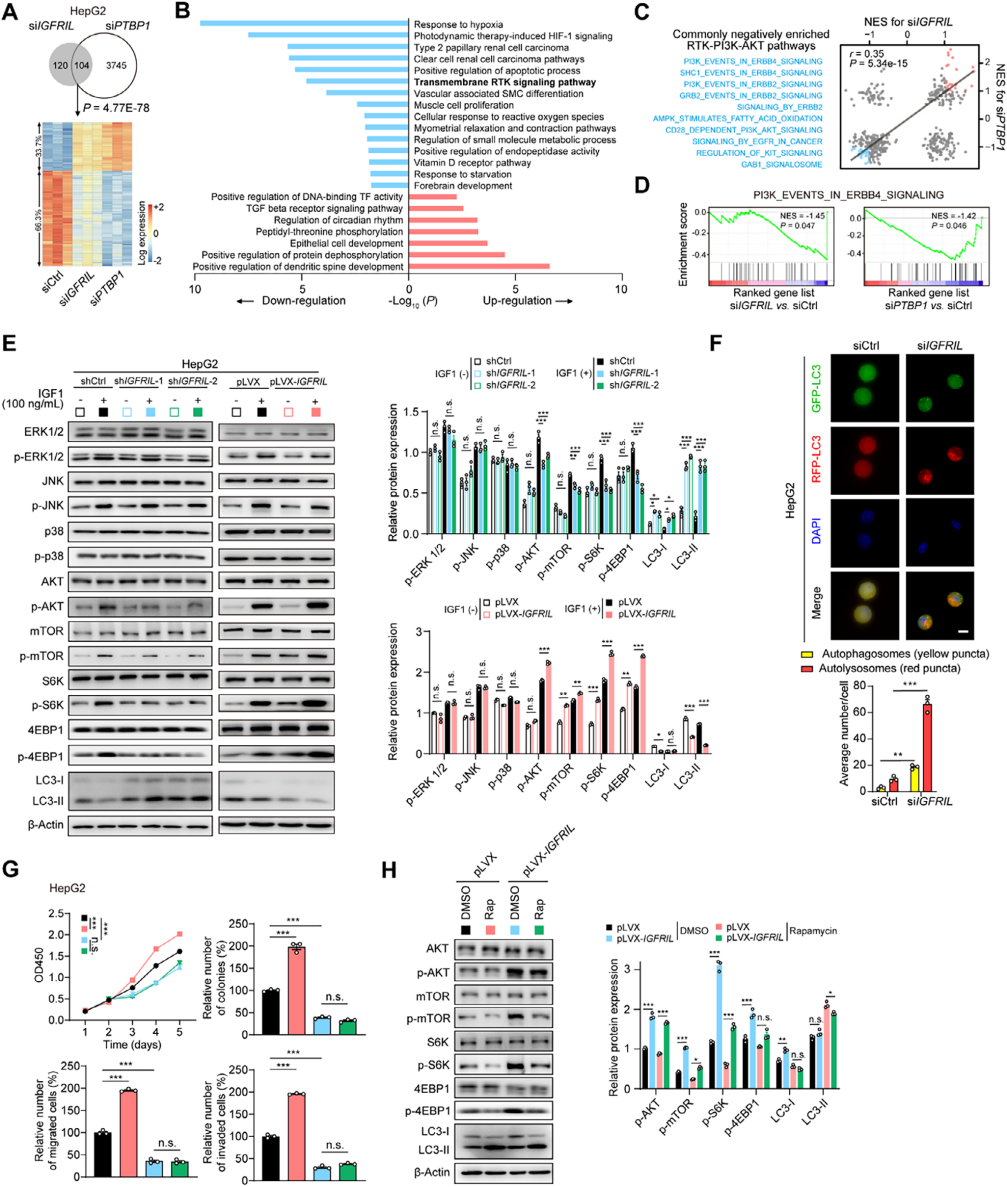
*IGFRIL* couples with PTBP1 to enhance the activation of AKT‐mTOR signaling in HCC cells. A) Overlapping DEGs upon *IGFRIL* and *PTBP1* knockdown in HepG2 cells. B) GO and KEGG pathway enrichment analyses of the overlapping differentially expressed genes (DEGs). C) Spearman's correlation of gene set enrichment scores between *IGFRIL* and *PTBP1* knockdown. NES, normalized enrichment score. D) GSEA plots of “PI3K_EVENTS_IN_ERBB4_SIGNALING” in *IGFRIL*‐knocked‐down and *PTBP1*‐knocked‐down HepG2 cells. E) IB assays of key cascades of the receptor tyrosine kinases (RTK) and AKT‐mTOR signaling in HepG2 cells upon *IGFRIL* knockdown or overexpression. F) Knockdown of *IGFRIL* accelerates the autophagic flux in HepG2 cells. HepG2 cells stably expressing mRFP‐GFP‐LC3 were treated with DMSO, NVP‐AEW541 (2 µm), or Rapamycin (10 µm) for 12 h upon knockdown of *IGFRIL*, followed by staining with DAPI. Red puncta represent the autophagosomes; and yellow puncta in the merged picture represent the autolysosomes. Scale bars, 40 µm. G,H) Rapamycin attenuates the promoting effects of *IGFRIL* overexpression on cell malignant phenotypes and mTOR signaling. Data are shown as mean ± SEM, ^*^ *P *< 0.05, ^**^ *P *< 0.01, ^***^ *P *< 0.001 by 1‐way ANOVA (E,G,H). n.s., not significant.

To identify shared targets of *IGFRIL* and PTBP1, we performed RNA‐seq analysis in *IGFRIL*‐ and *PTBP1*‐knockdown HepG2 cells. We observed a significant overlap in the differentially expressed genes (DEGS) between the two knockdown conditions (104 genes, *P* = 4.77E‐78; hypergeometric test; **Figure** **4**A, Table S6, Supporting Information), further supporting a functional link between *IGFRIL* and PTBP1. GO and KEGG pathway enrichment analyses of the overlapping genes revealed significant enrichment in the HIF‐1 signaling and receptor tyrosine kinase (RTK)‐associated gene sets (Figure 4B). Gene set enrichment analyses (GSEA) also showed high concordance in the enriched gene sets between *IGFRIL* and PTBP1 knockdown, particularly with a strong negative association with the RTK‐PI3K‐AKT signaling pathway (Figure 4C,D), a well‐known driver of cancer development.^[^ ^18^ ^]^

We then investigated the effect of *IGFRIL* on RTK signaling in HCC cells. We found that *IGFRIL* knockdown specifically inhibited AKT phosphorylation, while the phosphorylation levels of other RTK signaling components, such as ERK1/2, JNK, and p38, remained unchanged upon short‐ or long‐term stimulation with various RTK ligands (e.g., IGF1, insulin, PDGF, FGF2, and EGF) in HepG2 cells (Figure S5A, Supporting Information). Consequently, the phosphorylation of downstream cascades of AKT, including mTOR, S6K, and 4EBP1, was also reduced by *IGFRIL* knockdown in HepG2 and MHCC97H cells (Figure 4E; Figure S5B, Supporting Information). Conversely, overexpression of *IGFRIL* enhanced the phosphorylation of AKT, mTOR, S6K, and 4EBP1 in HepG2 and Huh7 cells (Figure 4E; Figure S5B, Supporting Information). These results indicate that *IGFRIL* specifically promotes the AKT‐mTOR pathway in HCC cells, without affecting other RTK signaling pathways.

Given that mTOR inhibition can induce autophagy with the conversion of LC3‐I to LC3‐II on autophagosomes,^[^ ^19^ ^]^ we hypothesized that *IGFRIL* might suppress autophagy through activation of the mTOR pathway. Indeed, *IGFRIL* knockdown increased LC3‐I to LC3‐II conversion, while *IGFRIL* overexpression decreased this conversion (Figure 4E; Figure S5B, Supporting Information). Furthermore, by measuring the fluorescence changes of tandem mRFP‐GFP‐LC3 constructs, which can distinguish autophagosomes from autolysosomes, we found that knockdown of *IGFRIL* in HepG2 cells leads to a marked increase in red fluorescence puncta (indicating autolysosomes) and a slight increase in yellow puncta (indicating autophagosomes), suggesting the acceleration of autophagic flux (Figure 4F). Similar results were observed in MHCC97H cells (Figure S5C, Supporting Information).

Similarly, PTBP1 also significantly activated the AKT‐mTOR pathway in HepG2, MHCC97H, and Huh7 cells (Figure S6A, Supporting Information). Additionally, PTBP1 was required for *IGFRIL*‐mediated activation of the AKT‐mTOR pathway and autophagy in HepG2 and Huh7 cells (Figure S6B, Supporting Information), further confirming the functional significance of their interaction. Finally, we observed that the promoting effects of *IGFRIL* overexpression on cell growth, migration, and invasion of HepG2 and Huh7 cells were abolished by mTOR inhibitor rapamycin (Figure 4G,H; S6C,D, Supporting Information). Collectively, these findings suggest that the *IGFRIL*‐PTBP1 complex plays the oncogenic roles dependent on the mTOR signaling.

### 2.5 *IGFRIL* Promotes the Binding of PTBP1 to *IGFBP3* mRNA to Reduce Its Expression

**Figure 5:**
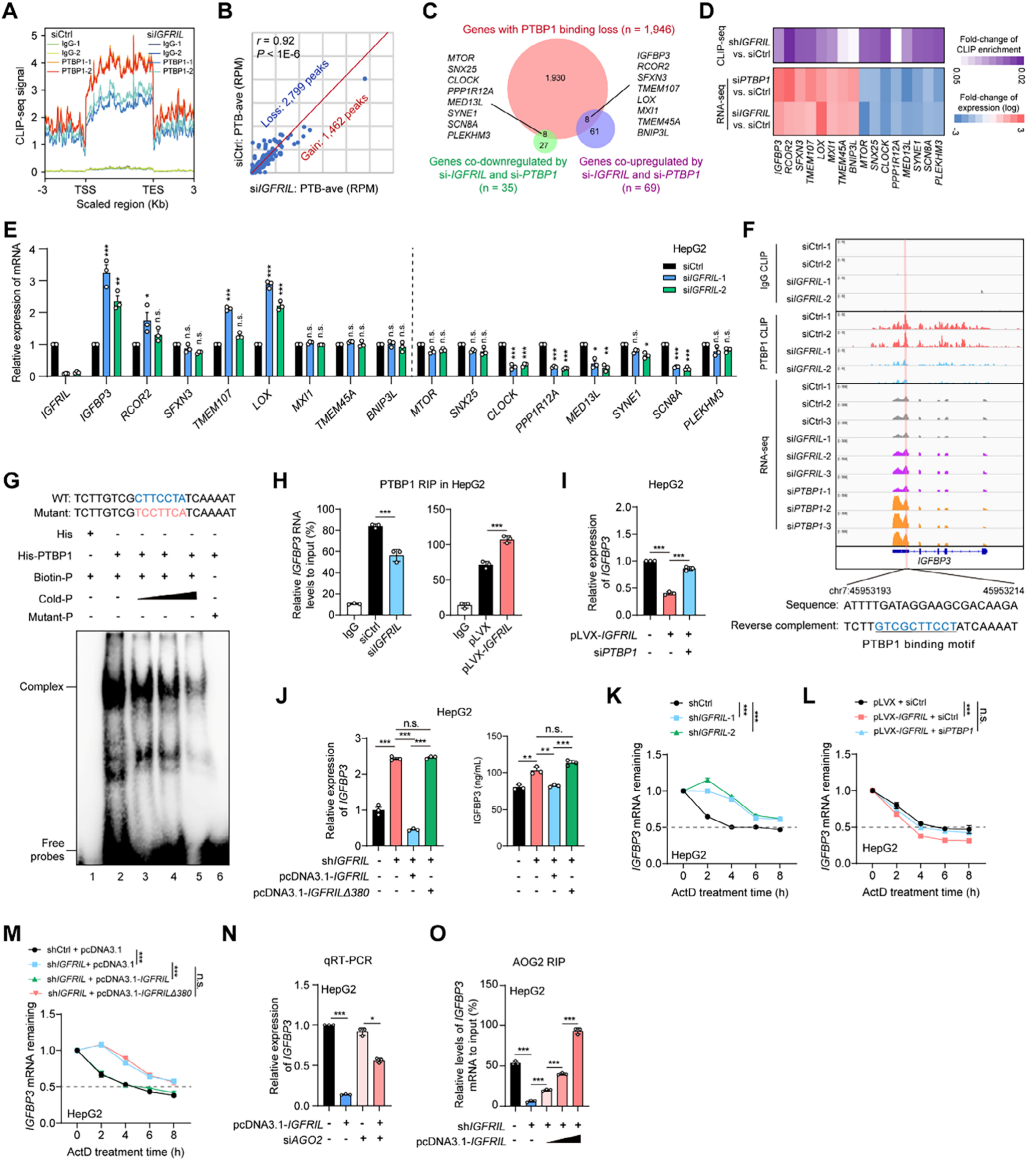
*IGFRIL* couples with PTBP1 to reduce the stability of *IGFBP3* mRNA. A) Distribution of PTBP1 binding sites across the transcriptome, showing altered binding patterns in HepG2 cells upon *IGFRIL* knockdown. TSS, transcriptional start site; TES, transcriptional end site. B) Pearson's correlation of the PTBP1 binding peak intensities determined by CLIP‐seq between groups. RPM, reads per million. C) Overlapping genes with altered PTBP1 binding and expression upon *IGFRIL* or *PTBP1* knockdown. D) Heatmap showing changes in PTBP1 binding and gene expression for 16 candidate genes. E) *IGFRIL* knockdown affects the mRNA expression of 16 candidate genes, including *IGFBP3*. F) Gene tracks showing the PTBP1 CLIP occupancies across the gene body of *IGFBP3* and the RNA abundance of *IGFBP3*. G) The binding of PTBP1 to the 3′ UTR of *IGFBP3* mRNA by electrophoresis mobility shift assay (EMSA). P, probe. H) *IGFRIL* enhances the interaction between PTBP1 and *IGFBP3* mRNA determined by RIP assays. I) *PTBP1* knockdown abolishes the *IGFRIL* overexpression‐induced repressive effect on *IGFBP3* mRNA. J) Re‐expression of full‐length *IGFRIL*, but not *IGFRIL△380*, reduces the promoting effect of *IGFRIL* knockdown on levels of mRNA and secreted protein of IGFBP3. K) The effects of *IGFRIL* knockdown on the stability of *IGFBP3* mRNA. L) The effects of *PTBP1* knockdown on the shortened stability of *IGFBP3* mRNA by *IGFRIL* overexpression. M) Re‐expression of full‐length *IGFRIL*, but not *IGFRIL△380*, re‐shortens the stability of *IGFBP3* mRNA in *IGFRIL*‐knocked‐down HepG2 cells. N) The effects of *IGFRIL* overexpression and *AGO2* knockdown on the mRNA levels of *IGFBP3*. O) Gradually increasing the re‐expressed *IGFRIL* in *IGFRIL*‐knocked‐down HepG2 cells restores the binding ability of AGO2 to *IGFBP3* mRNA by RIP assay. Data are shown as mean ± SEM, ^*^ *P *< 0.05, ^**^ *P *< 0.01, ^***^ *P *< 0.001 by 1‐way ANOVA (E,H,I,J,N,O) or repeated measures 1‐way ANOVA (K–M).

Next, we attempted to uncover the mechanism by which the *IGFRIL*‐PTBP1 complex activates the AKT‐mTOR pathway. The RNA‐binding protein PTBP1 is universally known to regulate mRNA stability and pre‐mRNA splicing. Therefore, we speculate that *IGFRIL* may affect the binding ability of PTBP1 to its target RNAs, thus regulating their stability and/or splicing. To this end, we first performed high‐throughput sequencing on RNAs isolated from HepG2 cells that bound to PTBP1 via cross‐linking immunoprecipitation (CLIP), in the presence or absence of *IGFRIL*. *IGFRIL* knockdown resulted in an overall decrease in PTBP1 binding across the transcriptome (**Figure** **5**A; Figure S7A, Supporting Information). While the distribution of PTBP1 CLIP peaks remained largely unchanged, *IGFRIL* knockdown led to an imbalance in PTBP1 binding affinity, resulting in the loss of binding signals at specific sites (Figure 5B; Figure S7C, Supporting Information). KEGG pathway enrichment analyses revealed that genes with increased PTBP1 CLIP signals after *IGFRIL* knockdown were primarily involved in protein and heterocycle catabolism, while those with decreased PTBP1 binding were involved in Rho GTPases, actin filament‐based processes, and cell cycle regulation (Figure S7D, Supporting Information).

To identify functional targets of the *IGFRIL*‐PTBP1 complex involved in RNA splicing and/or stability, we next integrated CLIP‐seq and RNA‐seq data from *IGFRIL*‐ and *PTBP1*‐knockdown HepG2 cells. Alternative splicing analysis revealed 104 overlapping dysregulated splicing events in 70 genes (Figure S7E, Table S6, Supporting Information). However, only 7 of these genes (e.g., *SPON2*, *ZBTB4*, *WDR43*, *BLM*, *LMF2*, *SF3B4*, and *CANX*) showed reduced PTBP1 occupancy upon *IGFRIL* knockdown, and none of them have been linked to the RTK‐AKT‐mTOR pathway. Therefore, we focused on genes with reduced PTBP1 occupancy and altered expression upon *IGFRIL* or *PTBP1* knockdown. This analysis identified a total of 16 genes (Figure 5C,D, Table S7, Supporting Information), of which the mRNA levels of 6 ones (including two negatively regulated genes *IGFBP3* and *LOX*, and four positively regulated genes *CLOCK*, *PPP1R12A*, *MED13L*, and *SCN8A*) were confirmed to be dependent on *IGFRIL* in both HepG2 and MHCC97H cells by qRT‐PCR assays (Figure 5E; Figure S7F, Supporting Information). Among these, IGFBP3 is the most abundant IGF‐binding protein and binds to IGF1 with an affinity comparable to or greater than IGF1R, thereby inhibiting IGF1R signaling and reducing AKT‐mTOR pathway activation.^[^ ^20^ ^]^ We therefore selected *IGFBP3* mRNA as a potential functional target of the *IGFRIL*‐PTBP1 complex for further investigation.

We identified multiple PTBP1‐binding sites on the *IGFBP3* pre‐mRNA, with the strongest site located in the 3′ UTR. This site exhibited a significant decrease in PTBP1 binding upon *IGFRIL* knockdown (Figure 5F). Electrophoresis mobility shift assay (EMSA) confirmed that PTBP1 binds to the predicted binding motif in the *IGFBP3* 3′ UTR (Figure 5G). RIP assays demonstrated that *IGFBP3* mRNA is enriched by PTBP1 antibody, and this enrichment is reduced by *IGFRIL* knockdown but enhanced by *IGFRIL* overexpression (Figure 5H), indicating that *IGFRIL* facilitates PTBP1 binding to *IGFBP3* mRNA. Accordingly, *IGFRIL* overexpression reduced *IGFBP3* mRNA and protein levels in HepG2 and Huh7 cells (Figure S7G, Supporting Information), and PTBP1 had a similar effect (Figure S7H,I, Supporting Information). Knockdown of *PTBP1* attenuated the inhibitory effect of *IGFRIL* overexpression on *IGFBP3* expression (Figure 5I; Figure S7J,K, Supporting Information). Consistently, the full‐length *IGFRIL*, but not the *IGFRIL*△380, reduced the promoting effects of *IGFRIL* knockdown on *IGFBP3* mRNA levels, as well as secreted protein levels determined by enzyme‐linked immunosorbent assays (ELISAs) (Figure 5J; Figure S7L,M, Supporting Information). Meanwhile, we found that *IGFRIL* knockdown also eliminated the weakened effect of PTBP1 overexpression on *IGFBP3* expression (Figure S7N,O, Supporting Information). Together, these findings demonstrate that *IGFRIL*, through its interaction with PTBP1, promotes the degradation of *IGFBP3* mRNA.

### 2.6 *IGFRIL* Enhances the PTBP1‐Mediated Instability of *IGFBP3* mRNA

To determine the mechanism by which the *IGFRIL*‐PTBP1 complex downregulates *IGFBP3* mRNA, we assessed the stability of *IGFBP3* mRNA using actinomycin D, which can effectively block *de novo* transcription. *IGFRIL* knockdown increased *IGFBP3* mRNA stability in HepG2 and MHCC97H cells, while *IGFRIL* overexpression decreased it in HepG2 and Huh7 cells (Figure 5K; Figure S8A, Supporting Information). PTBP1 played a similar role in reducing *IGFBP3* mRNA stability (Figure S8B, Supporting Information). Moreover, the inhibitory effect of *IGFRIL* overexpression on *IGFBP3* mRNA stability was abolished by *PTBP1* knockdown in HepG2 and Huh7 cells (Figure 5L; Figure S8C, Supporting Information). Consistently, the promoting effect of *IGFRIL* knockdown on *IGFBP3* mRNA stability was attenuated by overexpression of *IGFRIL*, but not *IGFRIL*△380 (Figure 5M; Figure S8D, Supporting Information). Additionally, qRT‐PCR analysis using exon junction‐specific primers for *IGFBP3* ruled out the possibility of *IGFRIL* affecting *IGFBP3* mRNA splicing (Figure S8E, Supporting Information). These findings suggest that the *IGFRIL*‐PTBP1 complex primarily regulates *IGFBP3* mRNA stability.

Further, given that PTBP1 has both local and long‐range effects on AGO2‐mediated mRNA stability,^[^ ^21^ ^]^ we explored whether the decreased *IGFBP3* mRNA stability by *IGFRIL*‐PTBP1 complex is dependent on AGO2. Indeed, knockdown of *AGO2* markedly attenuated the inhibitory effect of *IGFRIL* knockdown on *IGFBP3* levels in HepG2 cells (Figure 5N). Accordingly, RIP assays using the AGO2 antibody showed that knockdown of *IGFRIL* strongly reduces the binding of AGO2 to *IGFBP3* mRNA; while gradual increase of *IGFRIL* re‐expression in *IGFRIL*‐knocked‐down HepG2 cells can restore this binding in a dose‐dependent manner (Figure 5O). We also used cycloheximide (CHX) to block protein synthesis and assess the stability of IGFBP3 protein in HepG2 cells upon *IGFRIL* or *PTBP1* knockdown. No significant changes in IGFBP3 protein stability were observed (Figure S8F, Supporting Information), suggesting that PTBP1 does not directly affect protein stability. These findings indicate that the *IGFRIL*‐PTBP1 complex primarily regulates *IGFBP3* expression by destabilizing its mRNA.

### 2.7 *IGFRIL* Promotes the IGF1R‐AKT‐mTOR Axis and HCC Dependent on IGFBP3

**Figure 6:**
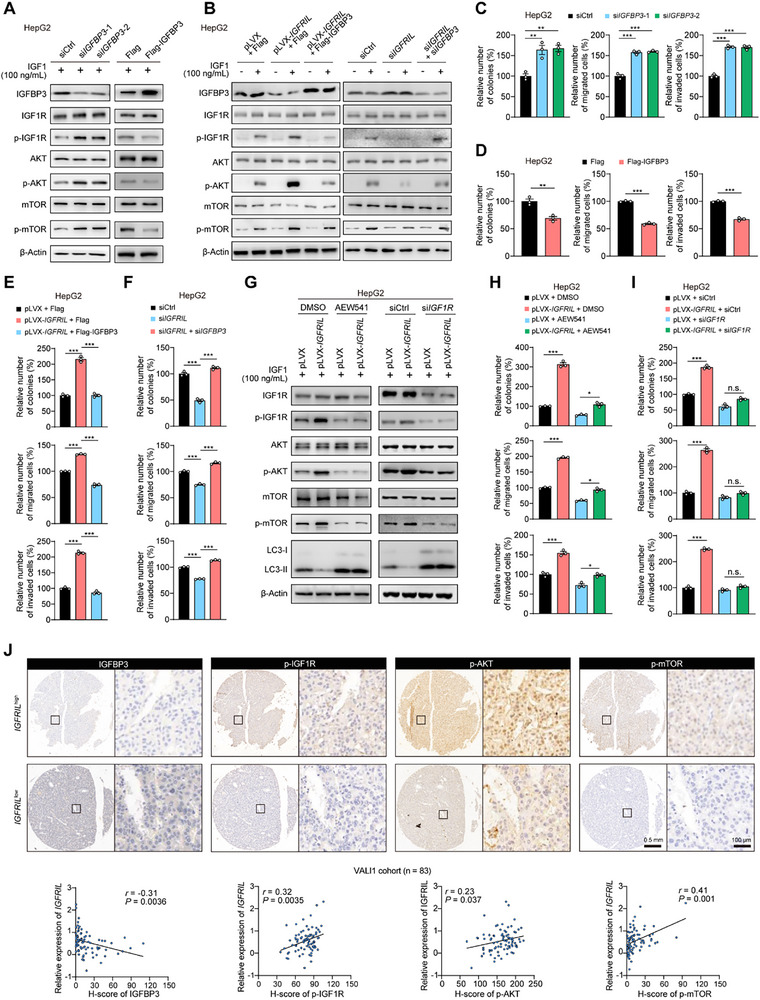
*IGFRIL* activates the IGF1R‐AKT‐mTOR signaling and plays oncogenic roles dependent on IGFBP3. A) The effects of knockdown or overexpression of *IGFBP3* on the activities of the IGF1R‐AKT‐mTOR axis. B) The effects of IGFBP3 overexpression on *IGFRIL*‐activated IGF1R‐AKT‐mTOR axis, and the effects of *IGFBP3* knockdown on *IGFRIL* knockdown‐repressed IGF1R‐AKT‐mTOR axis. C,D) The effects of *IGFBP3* knockdown or overexpression on the abilities of plate colony formation, migration, and invasion. E) The effects of IGFBP3 overexpression on the *IGFRIL*‐enhanced plate colony formation, migration, and invasion. F) The effects of *IGFBP3* knockdown on the *IGFRIL* knockdown‐reduced plate colony formation, migration, and invasion. G) The effects of IGF1R inhibition with its inhibitor AEW541 (2 µM) or siRNAs against *IGF1R* on the IGFRIL‐activated IGF1R‐AKT‐mTOR axis. H,I) The effects of IGF1R inhibition on the *IGFRIL*‐enhanced plate colony formation, migration, and invasion. J) j Spearman's correlations between the levels of *IGFRIL* and levels of IGFBP3, p‐IGF1R, p‐AKT and p‐mTOR, respectively, in HCC tumor tissues (*n* = 83) from the VALI1 cohort. Data are shown as mean ± SEM, ^*^ *P *< 0.05, ^**^ *P *< 0.01, ^***^ *P *< 0.001 by 1‐way ANOVA (C,E,F,G,I) or Student's *t‐*test (D).

Previous studies have shown that IGFBP3 suppresses IGF1R signaling in lung fibroblasts and breast cancer cells.^[^ ^22^, ^23^ ^]^ We found that *IGFBP3* knockdown increased IGF1R, AKT and mTOR phosphorylation in HepG2 and MHCC97H cells upon IGF1 stimulation, while IGFBP3 overexpression had the opposite effect in HepG2 and Huh7 cells (**Figure** **6**A; Figure S9A,B, Supporting Information). The enhanced phosphorylation of IGF1R, AKT, and mTOR induced by *IGFRIL* overexpression was reversed by IGFBP3 overexpression (Figure 6B; Figure S9C,D, Supporting Information). Conversely, the reduced phosphorylation of these proteins in *IGFRIL*‐knockdown cells was restored by *IGFBP3* knockdown (Figure 6B; Figure S9C,D, Supporting Information). Additionally, the *IGFRIL*‐dependent activation of IGF1R was also dependent on PTBP1 (Figure S9E, Supporting Information). Together, these findings indicate that the *IGFRIL*‐PTBP1 complex‐induced activation of the IGF1R‐AKT‐mTOR signaling is dependent on *IGFBP3* in HCC cells.

Next, we investigated the tumorigenic effect of IGFBP3 on HCC cells. *IGFBP3* knockdown increased cell proliferation, colony formation, migration, and invasion in HepG2 and MHCC97H cells (Figure 6C; Figure S9F,G, Supporting Information). Conversely, IGFBP3 overexpression decreased these cellular functions in HepG2 and Huh7 cells (Figure 6D; Figure S9H,I, Supporting Information). Furthermore, IGFBP3 overexpression attenuated the enhanced cell proliferation, colony formation, migration, and invasion induced by *IGFRIL* overexpression (Figure 6E; Figure S10A,B, Supporting Information). Conversely, *IGFBP3* knockdown rescued the reduced malignant phenotypes of *IGFRIL*‐knockdown cells (Figure 6F; Figure S10C,D, Supporting Information).

Furthermore, we observed that treatment with IGF1R inhibitor AEW541 or siRNAs targeting *IGF1R* weakens or nearly abolishes the promoting effect of *IGFRIL* overexpression on the levels of p‐AKT and p‐mTOR, and the LC3‐II: LC3‐I ratio in HepG2 and Huh7 cells (Figure 6G; Figure S10E, Supporting Information). *IGFRIL* overexpression attenuated serum starvation‐induced accumulation of autolysosomes and autophagosomes, which was marked by a significant reduction in red and yellow puncta. Interestingly, treatment with AEW541, Akti‐1/2, or Rapamycin not only further accelerated the starvation‐enhanced autophagic flux but also reversed the inhibitory effect of *IGFRIL* overexpression (Figure S10F, Supporting Information). Consistently, the promoting effect of *IGFRIL* overexpression on cellular phenotypes of HCC cells was largely weakened by AEW541 or siRNAs targeting *IGF1R* (Figure 6H,I; Figure S10G–J, Supporting Information).

Finally, immunohistochemistry (IHC) analysis of subcutaneous tumor tissues from nude mice showed that *IGFRIL* knockdown was associated with increased IGFBP3 and decreased p‐IGF1R, p‐AKT, and p‐mTOR levels (Figure S10K, Supporting Information). Combined IHC and qRT‐PCR analyses of human HCC tissues from the VALI1 cohort revealed a negative correlation between *IGFRIL* and IGFBP3 protein levels and a positive correlation between *IGFRIL* and the levels of p‐IGF1R, p‐AKT, and p‐mTOR (Figure 6J). These findings collectively suggest that *IGFRIL* activates the IGF1R‐AKT‐mTOR axis through PTBP1‐mediated destabilization of IGFBP3 mRNA, thereby promoting HCC tumorigenesis.

### 2.8 Upregulation of *IGFRIL* Sensitizes HCC Cells to IGF1R or mTOR Inhibitor

**Figure 7:**
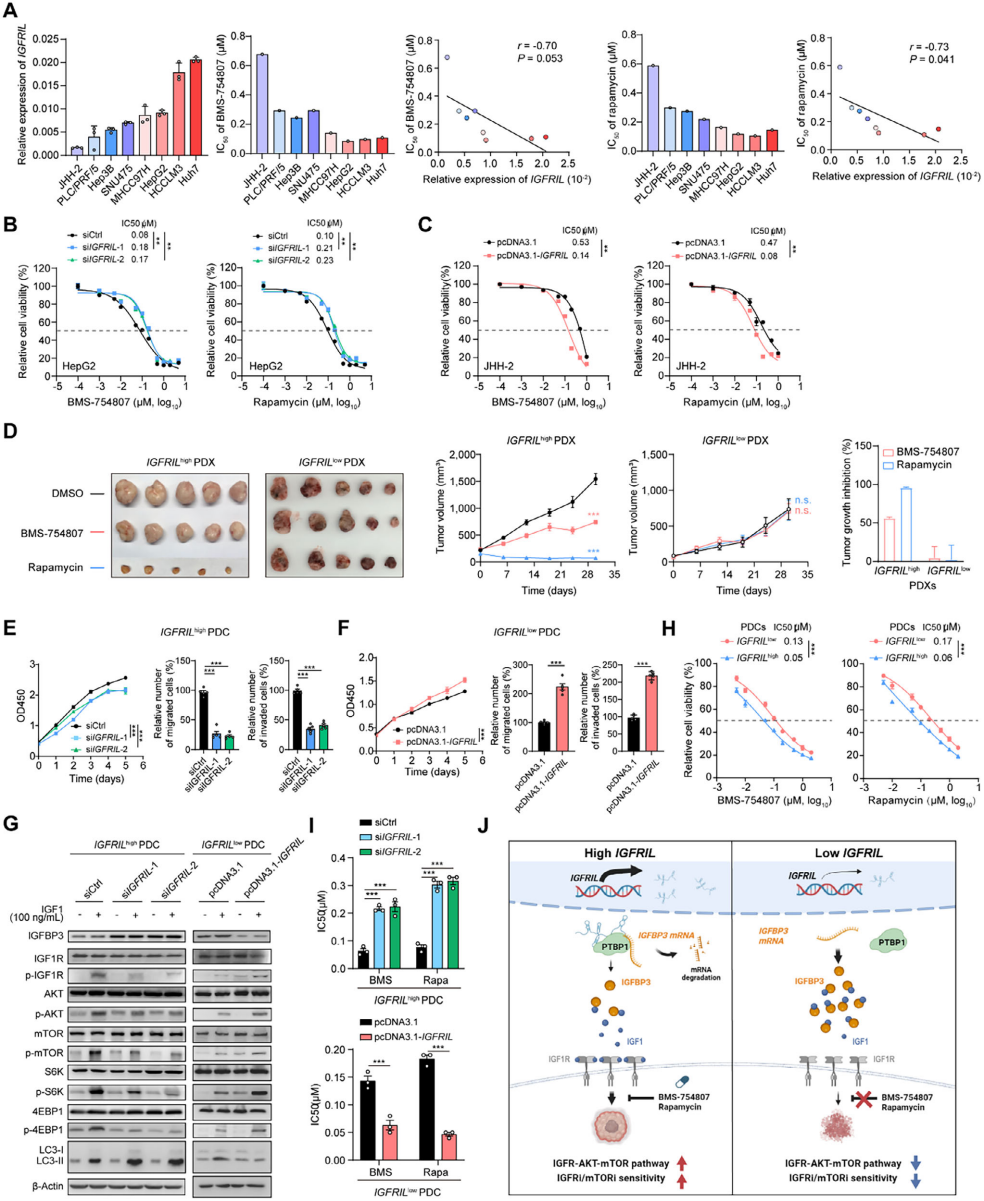
Upregulation of *IGFRIL* sensitizes HCC cells to IGF1R and mTOR inhibitors. A) Spearman's correlations between the levels of *IGFRIL* and sensitivities to inhibitors against IGF1R (BMS‐754807) and mTOR (rapamycin) in eight types of HCC cell lines. The effects of BMS‐754807 or rapamycin on HCC cell growth were determined by CCK‐8 assays. B) The effects of *IGFRIL* knockdown on the sensitivity to BMS‐754807 or rapamycin in HepG2 cells. C) The effects of *IGFRIL* overexpression on the sensitivity to BMS‐754807 or rapamycin in JHH‐2 cells. D) The effects of BMS‐754807 or rapamycin treatment on tumor growth in patient‐derived xenografts (PDX) mouse models. The mice were treated with control (DMSO), BMS‐754807 (25 mg kg^−1^ every 5 days, intraperitoneal injection) or rapamycin (10 mg kg^−1^ every 5 days, intraperitoneal injection) (*n* = 5 mice/group). E–G) The effects of *IGFRIL* knockdown or overexpression on the cell growth, migration, and invasion (E,F), and the activities of the IGF1R‐AKT‐mTOR axis (G) in *IGFRIL* ^high^ and *IGFRIL* ^low^ patient‐derived primary tumor cells (PDCs), respectively. H) The effects of BMS‐754807 (left) or rapamycin (right) treatment on the growth of *IGFRIL* ^high^ or *IGFRIL* ^low^ PDCs. I) The effects of *IGFRIL* knockdown or overexpression on the sensitivities to BMS‐754807 and rapamycin in *IGFRIL* ^high^ and *IGFRIL* ^low^ PDCs, respectively. J) Schematic diagram of the function and mechanism of *IGFRIL* in HCC tumorigenesis and drug sensitivity. Data are shown as mean ± SEM, ^*^ *P *< 0.05, ^**^ *P *< 0.01, ^***^ *P *< 0.001 by 1‐way ANOVA (B,D,E,I) or Student's *t* test (C,F,H).

Given the functional linkage between upregulation of *IGFRIL* and increased activity of the IGF1R‐AKT‐mTOR axis in HCC cells, we next speculate that upregulation of *IGFRIL* might sensitize the HCC cells to the inhibitors against IGF1R (IGF1Ri;, e.g., BMS‐754807) or mTOR (mTORi;, e.g., rapamycin). First, we observed that in a panel of HCC cell lines, those cells with higher levels of *IGFRIL* exhibit lower IGFBP3 levels (Figure S11A,B, Supporting Information) and half maximal inhibitory concentration (IC_50_) of BMS‐754807 or rapamycin than those cells with lower levels of *IGFRIL* (**Figure** **7**A). Furthermore, *IGFRIL* knockdown in *IGFRIL* ^high^ cells (e.g., HepG2 and MHCC97H) increased resistance to BMS‐754807 or rapamycin (Figure 7B; Figure S11C, Supporting Information). Conversely, *IGFRIL* overexpression in *IGFRIL* ^low^ cells (e.g., JHH‐2 and PLC/PRF/5) increased sensitivity to these inhibitors (Figure 7C; Figure S11D, Supporting Information).

We further investigated the effects of *IGFRIL* on drug sensitivity in HCC patient‐derived xenograft (PDX) models. Two PDX models were selected from our established 17 PDX models: *IGFRIL* ^high^ PDX 10 and *IGFRIL* ^low^ PDX 17 (Figure S11E, Supporting Information). Consistent with the oncogenic role of *IGFRIL*, the *IGFRIL* ^high^ PDX exhibited faster tumor growth, lower IGFBP3 levels, and higher levels of p‐IGF1R, p‐AKT, and p‐mTOR compared to the *IGFRIL* ^low^ PDX (Figure 7D; Figure S11E, Supporting Information). Treatment with BMS‐754807 or rapamycin significantly inhibited tumor growth in the *IGFRIL* ^high^ PDX model but had minimal effects on the *IGFRIL* ^low^ PDX model (Figure 7D). Of note, in the *IGFRIL* ^high^ PDX model, BMS‐754807 treatment significantly reduced the activity of the entire IGF1R‐AKT‐mTOR axis, and rapamycin treatment inhibited the mTOR activity. However, in the *IGFRIL* ^low^ PDX model, treatment with either drug had minimal effects on the phosphorylation of IGF1R, AKT, and mTOR (Figure S11F, Supporting Information).

We also assessed the effects of *IGFRIL* on drug sensitivity in cultured HCC patient‐derived primary tumor cells (PDCs) isolated from PDX models 10 (*IGFRIL* ^high^ PDC) and 17 (*IGFRIL* ^low^ PDC). Compared to *IGFRIL* ^low^ PDCs, *IGFRIL* ^high^ PDCs exhibited increased cell growth, migration and invasion (Figure 7E,F; Figure S11G,H, Supporting Information). *IGFRIL* knockdown in *IGFRIL* ^high^ PDCs reduced these malignant phenotypes, while *IGFRIL* overexpression in *IGFRIL* ^low^ PDCs increased them (Figure 7E,F; Figure S11G,H, Supporting Information). Furthermore, *IGFRIL* knockdown in *IGFRIL* ^high^ PDCs decreased the levels of p‐IGF1R, p‐AKT, p‐mTOR, p‐S6K, and p‐4EBP1, increased IGFBP3 levels and enhanced LC3‐I to LC3‐II conversion. Conversely, *IGFRIL* overexpression in *IGFRIL* ^low^ PDCs had the opposite effects (Figure 7G; Figure S11I, Supporting Information). Furthermore, we observed a consistent association between the endogenous *IGFRIL* levels and the sensitivity to BMS‐754807 or rapamycin in PDCs (Figure 7H). Notably, *IGFRIL* knockdown in the *IGFRIL* ^high^ PDCs reduced the sensitivity to BMS‐754807 or rapamycin (Figure 7I; Figure S11J, Supporting Information). Conversely, overexpression of *IGFRIL* sensitized the *IGFRIL* ^low^ PDCs to both inhibitors (Figure 7I; Figure S11K, Supporting Information). Collectively, these results indicate that the HCCs with high levels of *IGFRIL*, accompanied by high activity of the IGF1R‐AKT‐mTOR signaling, possess promising sensitivity to IGF1Ri or mTORi.

## 3 Discussion

LncRNAs are emerging as critical regulators of various human diseases, including cancer.^[^ ^24^ ^]^ Here, we identified a novel oncogenic lncRNA, *IGFRIL*, that is upregulated in HCC and associated with poor prognosis. Mechanistically, *IGFRIL* interacts with PTBP1 to destabilize *IGFBP3* mRNA, thereby activating the IGF1R‐AKT‐mTOR signaling pathway and promoting HCC development. Importantly, *IGFRIL* upregulation sensitizes HCC cells to IGF1R and mTOR inhibitors. Our findings highlight *IGFRIL* as a potential therapeutic target for HCC (Figure 7J).

PTBP1 is a multifunctional RBP involved in mRNA metabolism, including stability, splicing, polyadenylation, and translation initiation.^[^ ^16^, ^25^ ^]^ PTBP1 participates in various biological processes, such as neuronal differentiation, spermatogenesis, erythrocyte development, and cancer cell proliferation, migration, and senescence.^[^ ^26^, ^27^, ^28^ ^]^ LncRNAs can regulate RBP‐mediated mRNA metabolism by binding to RBPs. H19 and MEG3 are lncRNAs that regulate mRNA stability by recruiting PTBP1, contributing to hepatic steatosis and cholestatic liver injury.^[^ ^18^ ^]^ PTBP1‐mediated mRNA instability and alternative splicing have been implicated in various cancers. Here, we demonstrate that *IGFRIL* interacts with PTBP1 to enhance its binding to the *IGFBP3* 3′ UTR, promoting *IGFBP3* mRNA decay and activating the IGF1R‐AKT‐mTOR pathway. To our knowledge, *IGFRIL* is the first lncRNA identified to regulate PTBP1‐mediated RNA metabolism in HCC. Our findings provide insights into the complex interplay between lncRNAs and RBPs in cancer development.

The IGFR signaling pathway is frequently activated in HCC and contributes to tumor initiation and progression. IGFs are potent autocrine and paracrine mitogens, and their dysregulation has been linked to multiple cancers. As the key inhibitors of IGF1R signaling, IGFBPs, particularly IGFBP3, reduce the binding of IGF to IGF1R by competitively binding to IGF, thereby suppressing the activation of IGF1R signaling. There are many mechanisms for the downregulation of IGFBP3, including hypermethylation of the *IGFBP3* gene^[^ ^6^ ^]^ and proteolytic cleavage of the IGFBP3 protein;^[^ ^29^ ^]^ however, these mechanisms are not yet sufficient to fully explain the marked downregulation of IGFBP3 in tumors. Here, we revealed that *IGFRIL* reduces the stability of *IGFBP3* mRNA by coupling with PTBP1, and then induces the aberrant activation of the IGF1R‐AKT‐mTOR signaling in HCC. While previous studies have shown that PTBP1 can activate the AKT‐mTOR signaling via an alternative mechanism^[^ ^30^ ^]^ to our best knowledge, this study is the first to elucidate a novel post‐transcriptional regulatory mechanism involving the *IGFRIL*‐PTBP1 complex in IGFBP3‐mediated IGF1R‐AKT‐mTOR signaling. Our findings thus provide a crucial addition to the understanding of aberrant IGF1R‐AKT‐mTOR pathway activation in cancer.

Improved efficacy of anti‐HCC therapy might be achieved via the inhibition of the IGF1R‐AKT‐mTOR signaling in HCC cells.^[^ ^31^, ^32^, ^33^ ^]^ Although several IGF1Ri and monoclonal antibodies have shown promising activities in pre‐clinical studies of HCC,^[^ ^34^, ^35^, ^36^ ^]^ their clinical efficacy has been limited.^[^ ^37^ ^]^ Additionally, rapamycin and the first‐generation mTOR inhibitors have been used in clinical trials for the treatment of advanced HCC,^[^ ^38^ ^]^ or as adjuvant therapy for early‐stage HCC patients combined with transarterial chemoembolization (TACE) (ClinicalTrials ID: NCT02724332). However, only minimal efficacy was observed, suggesting the existence of primary resistance. Encouragingly, novel approaches selecting beneficiaries of the IGF1Ri and mTORi in stringently stratified patient cohorts are emerging as potential ways forward.^[^ ^39^, ^40^ ^]^ Here, we observed that high expression of *IGFRIL* facilitates the increased sensitivity to IGF1Ri or mTORi in HCC cell lines, pre‐clinical PDX and PDC models. However, dysregulation of *IGFRIL* does not alter the sensitivity of HCC cells to first‐line TKIs like sorafenib and lenvatinib (Figure S11L, Supporting Information). This may be due to these TKIs primarily targeting the Ras/Raf/MEK/ERK pathway rather than the PI3K/AKT pathway or other unexplored mechanisms. Therefore, it might be feasible to use IGF1Ri or mTORi to treat the HCC tumors bearing high *IGFRIL* expression. This idea of precise treatment suggests that it may be reasonable to choose “appropriate” HCC patients to take IGF1Ri or mTORi drugs, which certainly needs to be verified by future clinical trials. Additionally, our preliminary analyses showed that *IGFRIL* is also frequently upregulated in several other types of cancer and exhibits potential prognostic relevance (Figure 1F,G), indicating that *IGFRIL* is a novel trans‐cancer oncogenic lncRNA. Therefore, those types of cancer patients with high *IGFRIL* expression may also benefit from IGF1Ri or mTORi.

In summary, we identify a collection of novel HCC‐associated lncRNAs by transcriptome‐wide screening. Using an exemplary lncRNA *IGFRIL*, we reveal that it is a novel oncogene that potentiates the IGF1R signaling in the development of HCC. The discovery of *IGFRIL* may facilitate the development of precise treatment approaches for HCC. In addition to *IGFRIL*, it is also worth conducting in‐depth research on the other HCC‐associated lncRNAs identified in this study in the future.

## 4 Experimental Section

### Ethics Statements

Animals were housed in a specific pathogen‐free mouse facility at the animal center in the National Center for Protein Sciences, Beijing (NCPSB, China). The animal care and experimental protocols were approved (Approval number: IACUC‐DWZX‐2020‐799) by the Institutional Animal Care and Use Committee (IACUC) of NCPSB. All cancer patients and/or guardians obtain informed consent. Collection of the biopsy samples was performed according to the relevant ethical standards and was approved (Approval number: AF/SC‐08/02.93) by the Medical Ethical Committee of Beijing Institute of Radiation Medicine (Beijing, China).

### Patients and Tissue Specimens

This study included a total of three HCC cohorts (designated as VALI1, VALI2, and VALI3, respectively), one gastric cancer cohort (GC; designated as VALI4), and one colorectal cancer cohort (CRC; designated as VALI5). The VALI1 consisted of 124 HCC cases who were recruited from 2007 to 2016 at the Guangxi Cancer Hospital (Nanning City, China) and from 2009 to 2013 at the Jinling Hospital (Nanjing City, China); and their tumor tissues and matched adjacent non‐tumor liver tissues were collected. The VALI2 included 136 HCC cases recruited from the Sun Yat‐Sen University Cancer Center (Guangzhou City, China) from 2012 to 2013; and their tumor tissues and matched adjacent non‐tumor liver tissues were collected. The VALI3 consisted of 79 HCC cases recruited from 2014 to 2016 at the Sun Yat‐Sen University Cancer Center (Guangzhou City, China); and their tumor tissues and matched adjacent non‐tumor liver tissues were collected. The VALI4 included 96 gastric cancer cases who were recruited from 2012 to 2019 at Shandong Cancer Hospital (Jinan City, China); and their tumor tissues and matched adjacent non‐tumor gastric tissues were collected. The VALI5 consisted of 173 CRC cases who were recruited from the Sun Yat‐Sen University Cancer Center (Guangzhou City, China) from 2011 to 2012; and their 173 cancer tissues and 48 adjacent non‐tumor colorectal tissues were collected.

The diagnosis of HCC, the inclusion and exclusion criteria for HCC patients were described in detail previously.^[^ ^41^ ^]^ Briefly, the diagnosis of HCC was made by either positive histologic findings or an elevated serum α‐fetoprotein level (≥400 ng mL^−1^) combined with at least one positive image on angiography, sonography, and/or high‐resolution contrast computed tomography (CT). Gastric cancer patients were eligible on the basis of the following eligibility criteria: histologically confirmed cT3‐4aN + M0 gastric adenocarcinoma as detected by CT and/or magnetic resonance imaging according to the eighth Edition of the International Union against Cancer tumor‐node‐metastasis classification.^[^ ^42^ ^]^ Patients were excluded if they had a history of radiotherapy, chemotherapy, immunotherapy, or surgical treatment for gastric cancer and a history of other malignancies. The CRC cases were enrolled based on the following inclusion criteria: a clear pathological diagnosis, no prior treatment at the sampling point, and no history of other malignancies.^[^ ^43^, ^44^ ^]^ All pathologic data were reassessed and confirmed in consensus by two pathologists. This study was performed with the approval of the Medical Ethical Committee of Beijing Institute of Radiation Medicine (Beijing, China). At recruitment, informed consent was obtained from each patient, and personal information on demographic factors and clinical data was collected by a structured questionnaire. The detailed descriptions and summary of all cancer patients are provided in Table S1 (Supporting Information).

The RNAs in tissues from the VALI1 – VALI5 cohorts were used for RNA expression quantification by real‐time quantitative reverse transcription PCR (qRT‐PCR) assays. The proteins from the VALI1 cohort were also used for protein expression quantification by immunohistochemistry (IHC) assays (83 out of the 124 cases). Each specimen was reviewed by two board‐certified pathologists to confirm that the frozen sections were histologically consistent with tumor or non‐tumor tissues, and that tumor sections had to contain more than 70% of tumor cells. Tissues were kept frozen in liquid nitrogen immediately after separation until they were used for subsequent molecular studies.

### Cell Lines

The human HCC cell lines JHH‐2, HepG2 and HCCLM3 were obtained from the China Center for Type Culture Collection (CCTCC, Wuhan City, China); human HCC cell lines PLC/PRF/5 and SNU475 were generous gifts from Prof. Fuchu He (Beijing Institute of Lifeomics, Beijing, China); human HCC cell line MHCC97H was a generous gift from Prof. Shuhan Sun (Navy Military Medical University, Shanghai, China); human embryonic kidney cell line HEK293T, and HCC cell lines Hep3B and Huh7 were generous gifts from Prof. Chunyan Tian (Beijing Institute of Lifeomics, Beijing, China). All the cell lines were maintained in high‐glucose Dulbecco's modified Eagle's medium (DMEM; HyClone, USA) supplemented with 10% fetal bovine serum (FBS; HyClone, USA), 100 U mL^−1^ penicillin, and 100 µg mL^−1^ streptomycin. The cells were incubated at 37 °C in a humidified incubator containing 5% CO_2_.

### The 5′ and 3′ RACE Assays

The 5′ and 3′ rapid amplification of cDNA ends (RACE) cloning of *IGFRIL* was performed in HepG2 cells using SMARTerTM RACE cDNA amplification kit (Clontech, CA, USA) according to the manufacturer's instructions. The primers used for the PCR of the RACE assays are described in Table S8 (Supporting Information).

### RNA FISH and Immunofluorescence Assays

For determining the subcellular localization of *IGFRIL*, the specimens for RNA fluorescence in situ hybridization (FISH) and immunofluorescence (IF) assays were prepared as described previously.^[^ ^10^ ^]^ The *IGFRIL* probe was transcribed in vitro and was labeled using the DIG RNA labeling kit (SP6/T7; Roche, Switzerland). The primers for the generation of the *IGFRIL* probe are described in Table S8 (Supporting Information). The digoxigenin (DIG) haptens were detected by anti‐digoxigenin‐Rhodamine, Fab fragment (1:25 dilution; Roche, Switzerland). The nuclei were stained with 4′,6‐diamidino‐2‐phenylindole (DAPI). For assessing the autophagy activity in HCC cells, IF assays were performed as described previously.^[^ ^10^ ^]^ After treatment with chloroquine (CQ, 50 µm; s6999, Selleck, USA) or rapamycin (10 µm; s1039, Selleck, USA) for 12 h, a total of 1.2 × 10^5^ HCC cells were placed into confocal cells. Then, these HCC cells were washed twice in 1× PBS, fixed with 3% paraformaldehyde for 10 min, permeabilized with 0.5% Triton X‐100‐PBS for 5 min, and then blocked for 20 min using 5% horse serum dilution. Primary antibodies against LAMP (5% horse serum dilution; 1:200; 9091, CST, USA) and LC3 (5% horse serum dilution; 1:200; 83506, CST, USA) were then incubated with cells for 16 h at 4 °C. Then, the secondary antibodies conjugated with Alexa594‐379 (1:1000; Molecular Probes, USA) or Alexa488‐label (1:1000; Molecular Probes, USA) were incubated for 45 min at 37 °C. The nuclei were stained with 4′,6‐diamidino‐2‐phenylindole (DAPI). The signals were observed using a confocal laser‐scanning microscope LSM 700 (Carl Zeiss, Germany).

### Northern Blotting Assays

A total of 10 µg of the indicated RNAs was subjected to formaldehyde gel electrophoresis and transferred to Hybond N+ membranes. The *IGFRIL* probe was transcribed in vitro and was labeled using the DIG RNA labeling kit (SP6/T7; Roche, Switzerland). The primers for the generation of the *IGFRIL* probe are described in Table S8 (Supporting Information). After 60 min of prehybridization in buffer A consisting of 20 µg mL^−1^ fish sperm DNA (fsDNA), 50 µg mL^−1^ heparin, 50% Deionized formamide, 0.1% SDS, 5× Denhardt's, and 5× SSC (PH = 7), the membrane was hybridized for 12 h at 68 °C in buffer A containing the denatured probe. The membrane was washed using buffer B containing 2× SSC and 0.1% SDS. Then, the membrane was incubated with anti‐digoxigenin‐AP, Fab fragment (1:3000 dilution; Roche, Switzerland) conjugated to alkaline phosphatase (AP) for 1 h at home temperature. After the addition of AP substrate, the immunoreactive bands were detected by NBT/BCIP kit (CWBIO, China) according to the manufacturer's instructions.

### Knockdown or Overexpression of Genes

The eukaryotic expression vector pcDNA3.1 (+) (Invitrogen, USA) was used for overexpression of *IGFRIL*, *PTBP1*, or *IGFBP3*. The intact cDNA sequences of *PTBP1* (NM_002819.5) and *IGFBP3* (NM_001013398.2) were synthesized by Biomed Co., (Beijing, China) and cloned into the vectors. Then, the empty vectors or the vectors containing the intact cDNAs of these three genes were transfected into HCC cells using Lipofectamine 2000 (Invitrogen, USA). The siRNAs targeting *IGFRIL*, *PTBP1*, *IGFBP3*, or *IGF1R* genes, and the scrambled control siRNAs were synthesized by Ribobio Co., (Guangzhou City, China). RiboFECT CP transfection kit (Ribobio, China) and Lipofectamine 2000 reagent (Invitrogen, USA) were used for transfection of siRNAs and plasmids, respectively, according to the manufacturer's instructions. To stably knock down the expression of *IGFRIL* and *PTBP1* genes, specific shRNAs targeting *IGFRIL* or *PTBP1* and a non‐targeting scrambled control shRNA were designed and synthesized by Inovogen Co., (Beijing, China). These shRNAs were cloned into the pLVX‐shRNA‐Puro vector system. The vectors with shRNAs along with viral packaging plasmids (PMD and SPA) were transfected into HEK293T cells using Lipofectamine 2000 (Invitrogen, USA). Virus supernatant was then harvested after 48 h, filtered through a 0.45‐µm filter, and incubated with target cells for 8 h at a 1:10 dilution with 8 µg mL^−1^ polybrene. Infected cells were then selected with 200 µg mL^−1^ puromycin for 2 weeks before evaluation for efficiency of gene knockdown. All the media contained 10% FBS, 100 U mL^−1^ penicillin, and 100 mg mL^−1^ streptomycin (15140‐122; Gibco, USA).

### Cell Growth, Plate Colony Formation, Migration, and Invasion Assays

The growth of HCC cells was evaluated using the CCK‐8 kit (CK04, Dojindo, Japan) in accordance with the manufacturer's instructions. Briefly, a total of 2 × 10^3^ well^−1^ of HCC cells were seeded in 96‐well plates. Subsequently, 10% CCK‐8 reagent was added to each well at five‐time points (days 1, 2, 3, 4, and 5), followed by incubation at 37 °C for 90 min. The optional density (OD) was measured at 450 nm using a spectrophotometer. For plate colony formation assays, a total of 300 cells (for assessing the pro‐tumoral effects in overexpression assays) or 600 cells (for assessing the anti‐tumoral effects in knockdown assays) were plated in six‐well plates and cultured for 2 weeks. Then, cells were washed twice with phosphate‐buffered saline (PBS), fixed with 4% paraformaldehyde for 30 min, and then stained with crystal violet staining solution (Solarbio, China) for 10 min. The visible colonies were then photographed and counted. For migration and invasion assays, a total of 7 × 10^4^ cells (200 µL; for assessing the pro‐tumoral effects in overexpression assays) or 1.4 × 10^5^ cells (200 µL; for assessing the anti‐tumoral effects in knockdown assays) were planted on the top chamber of each insert (Cat. 353097; BD Biosciences, USA) or matrigel invasion chamber (Cat. 354480; BD Biosciences, USA). Then, 800 µL DMEM supplemented with 20% FBS was injected into the lower chambers. After incubation at 37 °C for 24–48 h, the cells adhering to the lower side of the inserts were stained with 0.5% crystal violet solution, and then imaged and counted by IX71 inverted microscope (Olympus, Japan). Cell numbers in three random fields were counted. All experiments were performed at least three times.

### RNA Extraction and qRT‐PCR Assays

Total RNAs from HCC cells and tissues were isolated using the RNA purification kits (CW0584S; CWBIO, China), and genomic DNAs (gDNAs) from HCC cells were extracted using a genomic DNA extraction kit (Tiangen, Beijing, China), according to the manufacturer's instructions. Polyadenylated RNAs were captured by oligo (dT) polystyrene beads using GenElute 234 TM mRNA miniprep kit (Sigma‐Aldrich, USA) and purified according to the instructions. The cytoplasmic and nuclear fractions of cells were isolated using the Cytoplasmic and Nuclear RNA Purification Kit (Cat. 37400; Norgen, USA). The cDNAs were synthesized from 500 ng of total RNA using PrimeScript RT reagent kit (RR037A; Takara, Japan) according to the manufacturer's protocol. The real‐time quantitative reverse transcription PCR (qRT‐qPCR) assays were performed using SYBR FAST qPCR kit (KK4607; Kapa Biosystems, USA) on IQ5 real‐time PCR system (BioRad, USA). The relative expression levels of RNAs were calculated using the comparative C_t_ method. Melting curve analyses were performed on all PCRs to rule out non‐specific amplification. *GAPDH* and *U6* were used as the internal controls, and each sample was performed in triplicate. For quantifying the abundance of nuclear and cytoplasmic *IGFRIL*, *U6* (nuclear marker) and *GAPDH* (cytoplasmic markers) were used as positive controls, respectively. For the determination of the polyadenylated *IGFRIL*, *ACTB*, and *H19* (polyadenylated RNAs) were used as positive controls, while *18S rRNA* (non‐polyadenylated RNA) was used as a negative control. All primers used for qRT‐PCR assays are listed in Table S8 (Supporting Information).

### Immunoblotting Assays

After 24 h of serum starvation, HCC cells were treated with IGF1 (100 ng mL^−1^; C032, Novoprotein, China), insulin (100 ng mL^−1^; HY‐P73243, MedChemExpress, USA), PDGF (100 ng mL^−1^; HY‐P7055, MedChemExpress, USA), EGF (100 ng mL^−1^; HY‐P7109, MedChemExpress, USA) or FGF2 (100 ng mL^−1^; HY‐P7004, MedChemExpress, USA) for 15 or 30 min, and then subjected to immunoblotting assays for indicated proteins. To examine the involvement of mTOR signaling in *IGFRIL*’s oncogenic role, HCC cells were treated with the mTOR activator MHY1485 (10 µm; s7811, Selleck, USA) or inhibitor rapamycin (5 µm; s1039, Selleck, USA) for 1 h. Cells were harvested and lysed in RIPA lysis buffer (CW2333S; CWBIO, China) containing protease inhibitor cocktail (1:100; 04693132001, Roche, Switzerland) and phosphatase inhibitor cocktail (1:100; CW2383S, CWBIO, China) for 30 min. Total proteins (10–20 µg) were re‐suspended in Laemmli buffer (63 mm Tris‐HCl, 10% glycerol, 2% SDS, and 0.0025% bromophenolblue; pH = 6.8) and electrophoresed on SDS‐polyacrylamide gels. Then, proteins were transferred to a polyvinylidene difluoride membrane. Membranes were incubated with the indicated primary antibodies and anti‐mouse or anti‐rabbit secondary antibodies conjugated to horseradish peroxidase (HRP). The immunoreactive bands were detected using the SuperSignal West Pico chemiluminescent substrate kit (34577; Thermo, USA) on an immunoblotting detection system (TANON, China).

The following primary antibodies were used: anti‐PTBP1 (1:1000; 32–4800, Thermo, USA), anti‐GAPDH (1:1000; 60004‐1‐Ig, Proteintech, USA), anti‐β‐actin (1:1000; ab8227, Abcam, USA), anti‐ERK1/2 (1:500; 4696, CST, USA), anti‐p‐ERK1/2 (1:500; 4376, CST, USA), anti‐JNK (1:500; 9252, CST, USA), anti‐p‐JNK (1:500; 9251, CST, USA), anti‐p38 (1:500; 9212, CST, USA), anti‐p‐p38 (1:500; 9215, CST, USA), anti‐AKT (1:500; 10176‐2‐AP, Proteintech, USA), anti‐p‐AKT (1:500; 4060, CST, USA), anti‐mTOR (1:500; 2972, CST, USA), anti‐p‐mTOR (1:500; 2976, CST, USA), anti‐S6K (1:500; 2708, CST, USA), anti‐p‐S6K (1:500; 9234, CST, USA), anti‐4EBP1 (1:500; 9644, CST, USA), anti‐p‐4EBP1 (1:500; 9455, CST, USA), anti‐LC3 (1:500; 4108, CST, USA), anti‐IGFBP3 (1:500; 10189‐2‐AP, Proteintech, USA), anti‐IGF1R (1:500; 3027, CST, USA) and anti‐p‐IGF1R (1:500; 3024, CST, USA).

### RNA Pull‐Down Assays and Mass Spectrometry

The cDNAs encoding *IGFRIL*, antisense‐*IGFRIL*, truncated versions of *IGFRIL*, or *GAPDH* were amplified by PCR and subcloned into the pGEMT vectors for in vitro transcription. Then, the biotin‐labeled RNA transcripts were produced in vitro using transcription optimized 5× buffer (Promega, USA), biotin RNA labeling mix (Roche, Switzerland), and T7 RNA polymerase (Roche, Switzerland), treated with RNase‐free DNase I (Roche, Switzerland), and purified using the RNeasy mini kit (Qiagen, USA). A total of 3 µg biotinylated RNAs were mixed with proteins extracted from HepG2 cells, followed by targeting RNAs using streptavidin beads (Millipore, USA). The co‐precipitated proteins were then subjected to SDS‐PAGE or mass spectrometry (MS) analyses, and further visualized by silver staining or immunoblotting assays.

For MS analyses, gel bands were minced and destained with 50% acetonitrile in 50 mm ammonium bicarbonate. Proteins were reduced with 10 mm dithiothreitol (DTT) at 56 °C, followed by alkylation with 55 mm iodoacetamide at room temperature in the dark. Trypsin digestion was performed overnight at 37 °C with gentle shaking. Peptides were extracted using 1% trifluoroacetic acid in 50% acetonitrile. Samples were vacuum‐dried and reconstituted in 0.1% formic acid for subsequent MS analysis. MS analysis was then performed using an Easy‐nLC 1200 UHPLC coupled to a QExactive HFX mass spectrometer (Thermo Fisher Scientific, MA, USA). The tandem mass spectra were searched against the human UniProt database (Version 20140922; including 20193 sequences) using MaxQuant (Version 1.5.3.30).^[^ ^45^ ^]^ The database search was conducted with the following parameters: 1) Peptides with a minimum length of 7 amino acids were retained; 2) Trypsin was selected as the proteolytic enzyme with up to two missed cleavages sites allowed; 3) Variable modifications included the oxidation of methionine (M) residues and acetylation of the protein N‐terminal; 4) The first search mass tolerance and main search peptide tolerance were set to 20 and 4.5 ppm, respectively; 5) The FDRs of the peptide‐spectrum matches and proteins were set to less than 1%; and6) At least two matching unique peptides were required to obtain reliable protein identification.

### RNA Immunoprecipitation (RIP) Assays

RIP assays were performed using the Magna RIP RNA‐Binding Protein Immunoprecipitation kit (Millipore, USA). Antibody against PTBP1 (32‐4800; Thermo Fisher, USA) or against AGO2 (Cat. 2897; CST, USA) was used for the RIP. Cells were cultured in a 10‐cm plate, and then washed twice using 10 mL ice‐cold PBS, scraped off in 10 mL PBS, and collected by centrifugation at 1500 revolutions per min (rpm) for 5 min at 4 °C. The cell pellets were re‐suspended in an equal pellet volume of complete RIP lysis buffer. One hundred µL lysis and beads were incubated in 900 µL immunoprecipitation buffer overnight at 4 °C. Beads were collected by magnetic separator and washed by RIP wash buffer by five times with shaking. The immunoprecipitates were re‐suspended in 150 µL proteinase K buffer and incubated at 55 °C for 30 min with shaking. After incubation, the supernatants were transferred into 250 µL of RIP wash buffer and 400 µL of phenol: isoamyl alcohol to each tube. After centrifugation, the aqueous phase was removed in a new tube, mixed with 400 µL chloroform. Salt solution was added to enhance the precipitation of RNA at −80 °C for overnight. After centrifugation, the pellets were washed by 80% ethanol and re‐suspended in 15 µL of RNase‐free water, and the isolated RNAs were used for subsequent qRT‐PCR assays.

### Autophagic Flux Measurement

Autophagic flux was monitored by transfection with the tandem mRFP‐GFP‐LC3 constructs. HepG2 and MHCC‐97H cells were cultured in a six‐well plate for 24 h and subsequently transfected with the mRFP‐GFP‐LC3 lentivirus (HBLV‐1011, HANBIO, China) for 8–10 h. After 24–48 h of culture, infected cells were selected with 200 µg mL^−1^ puromycin for 2 weeks, and then the efficiency of RFP and GFP expression was evaluated. For the knockdown and overexpression assays, si*IGFRIL* and pcDNA3‐*IGFRIL* were separately transfected into mRFP‐GFP‐LC3‐positive cells and their respective control groups of cells. After treatment with inhibitors targeting the IGF1R‐AKT‐mTOR pathway for 12 h, including NVP‐AEW541 (2 µm; S1034, Selleck, USA), Rapamycin (10 µm; S1039, Selleck, USA), and Akti‐1/2 (5 µm; S7776, Selleck, USA), cell nuclei were stained with Hoechst 33342 (10 µg mL^−1^; C0031, Solarbio, China). Fluorescence images were captured using a Confocal (Olympus, Tokyo, Japan) for the detection of autophagosomes (yellow puncta in fusion images) and autolysosomes (red puncta in fusion images). The number of YFP and RFP dots was determined by manually counting the fluorescent puncta.

### Electrophoretic Mobility Shift Assay (EMSA)

Purified PTBP1 and biotin‐labeled RNA probes (a 22‐nt fragment of the 3′ UTR of *IGFBP3* containing the binding motif of PTBP1) were incubated in binding buffer at room temperature for 4 h. Serial dilutions of unlabeled wild‐type probes or mutated probes were added to the reaction for competition assays. Samples were diluted with loading buffer and loaded onto a native polyacrylamide gel. Electrophoresis was performed at 80 V for 1 h, followed by transferring to a nylon membrane at 80 V for 1 h with a transfer unit (BioRad, USA). After transfer, RNAs were immobilized with a UV cross‐linker and dried at 42 °C for 15 min. The membrane was blocked with blocking buffer (11096176001; Roche, Switzerland) for 30 min at room temperature, and then incubated with Streptavidin‐HRP (LI 925–32230; LI‐COR Biosciences, USA) for 45 min. The membrane was washed three times with washing buffer and then imaged by the Odyssey Infrared Imaging System (LI‐COR Biosciences, USA). The probes are listed in Table S8 (Supporting Information).

### Determination of Stability of *IGFBP3* mRNAs and Proteins

To examine the stability of *IGFBP3* mRNAs in HCC cells, HepG2 or MHCC97H cells were treated with actinomycin D (Act.D; 5 µg mL^−1^; APExBIO, USA) for 2, 4, 6, and 8 h, respectively, and then the remaining *IGFBP3* mRNAs at each time point were quantified by qRT‐PCR assays and normalized to the *IGFBP3* mRNA levels in the untreated cells. To examine the stability of IGFBP3 protein in HCC cells, HepG2 cells were treated with cycloheximide (CHX; 100 µg mL^−1^; Cat. 2112, CST, USA) for 0.5, 1, 2, 3, and 5 h, respectively, and then the remaining IGFBP3 proteins at each time point were quantified by immunoblotting assays and normalized to the IGFBP3 protein levels in the untreated cells.

### Enzyme‐Linked Immunosorbent Assay (ELISA)

The supernatants of cultured HCC cells after starvation were collected, and IGFBP3 levels were assessed using an IGFBP3 ELISA kit (Cat. 64143; CST, USA) according to the manufacturer's instructions. For all ELISA experiments, each sample was assayed in triplicate, and the absorbance was measured using a Tecan's Sunrise microplate reader (Tecan Sunrise, Switzerland).

### Immunohistochemistry (IHC) Assays

HCC tissues were fixed in 4% paraformaldehyde, embedded in paraffin, and sectioned. Sections were then dewaxed in xylene and rehydrated in different grades of ethanol. After antigen retrieval in EDTA, sections were incubated with the primary antibody overnight at 4 °C and then incubated with secondary antibody for 2 h at room temperature. The primary antibodies used for IHC assays in this study included: anti‐Ki‐67 (1:200; sc‐23900; Santa Cruz, USA), anti‐IGFBP3 (1:200; 64143, CST, USA), anti‐p‐IGF1R (1:800; 3021, CST, USA), anti‐p‐AKT (1:500; 9611, CST, USA) and anti‐p‐mTOR (1:100; 2976, CST, USA). Then, the sections were stained with DAB and hematoxylin. Images were taken with a microscope (Olympus, Japan). Briefly, the IHC signals in clinical specimens were detected on a light microscope, and the pixelwise H‐score was evaluated based on the assessment incorporating both the staining intensity and the percentage of stained cells (*P*) at each intensity level. The final H‐score was derived from the sum of intensity multiplied by *P*, which was in the range of 0 to 300. The protein expression levels in tumors derived from mouse models were determined semi‐quantitatively according to the percentage of positively stained cells and the staining intensity as previously described.^[^ ^41^ ^]^ Briefly, a proportion score was assigned representing the estimated proportion of positive staining tumor cells (0, none; 1, <1/100; 2, 1/100 to <1/10; 3, 1/10 to <1/3; 4, 1/3 – 2/3; and 5, >2/3). The average estimated intensity of staining in positive cells was assigned an intensity score (0, none; 1, +; 2, ++; 3, +++; and 4, ++++). The overall scores (0 or 2–9) were obtained by combining these two parameters.

### Nude Mice Studies

Four‐week‐old male athymic nude BALB/c mice were purchased from the Vital River Laboratories (Beijing, China). All mice were maintained in a specific‐pathogen‐free (SPF) facility, in accordance with a protocol approved by the Animal Ethics Committee of the National Center for Protein Sciences, Beijing (NCPSB, China).

To assess the effects of *IGFRIL* on tumor growth in vivo, four‐week‐old male athymic nude mice (BALB/c background) were randomly divided into the indicated groups before inoculation. A total of 3 × 10⁶ HepG2 cells transfected with shCtrl, sh*IGFRIL*‐1 or sh*IGFRIL*‐2, or 2 × 10⁶ HepG2 cells transfected with pLVX or pLVX‐*IGFRIL* were subcutaneously injected into the left or right flanks of the mice (*n* = 10/group). Tumor growth was recorded every 7 days with a calliper and tumor volume was calculated as *a* × *b* ^2^ × 0.5 (a, the longest diameter; and b, the shortest diameter). Tumors were allowed to grow for 4–5 weeks. All the mice were sacrificed, and the tumors were picked out. Tumor weights were measured. All tumors were fixed with paraformaldehyde (4%) before dehydration and embedded in paraffin. Paraffin sections were used for hematoxylin and eosin (H&E) staining, as well as for IHC assays of Ki‐67, IGFBP3, p‐IGF1R, p‐AKT, and p‐mTOR.

For metastasis assays, HepG2 or MHCC97H cells bearing firefly luciferase were stably transfected with the indicated vectors (pLVX vs pLVX‐*IGFRIL*, or shCtrl vs sh*IGFRIL*‐1/sh*IGFRIL*‐2, respectively). Cells were dissociated with trypsin, washed with PBS, and brought to a concentration of 1 × 10^6^ cells mL^−1^. These cells were intravenously injected into the tail veins of nude mice (*n* = 10/group). Lung metastatic signals were detected using the IVIS system (124264; Xenogen, USA). The mice were sacrificed after 4 weeks of injection, and the lungs were excised and then fixed in 4% paraformaldehyde. Five non‐sequential serial sections per lung were obtained. The sections were stained with H&E and analyzed for metastases by light microscopy (Leica, Germany). The total number of metastases per lung section was counted and averaged among the mice.

### Drug Sensitivity Testing—In Vitro Assays in HCC Cell Lines

Cells were plated in 100 µL of complete growth medium at a density of 2 × 10^3^ well^−1^ in 96‐well tissue culture plates. After serial dilutions, 100 µL of growth medium containing BMS‐754807 (Cat. S1124; Selleck, USA), rapamycin (Cat. S1039; Selleck, USA), sorafenib (Cat. S7397; Selleck, USA), or lenvatinib (Cat. E7080; Selleck, USA) was added to cells. Meanwhile, dimethyl sulfoxide (DMSO) was used as a solvent control. Cell viability was determined using the CCK‐8 assays and normalized to the DMSO control group and was expressed as a percentage of maximum proliferation. The half maximal inhibitory concentration (IC_50_) values were generated and compared using GraphPad Prism (version 9).

### Drug Sensitivity Testing—In Vivo Assays in Patient‐Derived Tumor Xenograft (PDX) Mouse Models

A total of 17 HCC patients were recruited from the Fifth Medical Center of Chinese PLA General Hospital (Beijing, China), and the tumor tissues were subjected to the establishment of PDX mice models by IDMO Co., Ltd. (Beijing, China). Based on the *IGFRIL* levels measured by qRT‐PCR assays in these tumors, two tumors were selected for further study, one with high endogenous *IGFRIL* levels (*IGFRIL* ^high^) and the other with low endogenous *IGFRIL* levels (*IGFRIL* ^low^). All experiments were approved by the Fifth Medical center of the Chinese PLA General Hospital. Briefly, fresh tumor tissues were placed in ice‐chilled high‐glucose DMEM with 10% FBS, 100 U mL^−1^ penicillin, and 100 U mL^−1^ streptomycin and rapidly processed for engraftment. After removal of necrotic tissues, the tumors were partitioned into 2 × 1 × 1 mm^3^ blocks and washed 3 times with ice‐cold PBS. The tissue blocks were incubated in DMEM medium supplemented with 50% Matrigel (356 234; BD, USA), 10 ng mL^−1^ epidermal growth factor (PHG0314; Gibco, USA), 10 ng mL^−1^ basic fibroblast growth factor (PHG0264; Gibco, USA), 100 U mL^−1^ penicillin, and 100 U mL^−1^ streptomycin for 30 min. Three blocks of tumor tissues with the incubation mix (Matrigel plus growth factors) were subcutaneously transplanted into the right flanks of 5‐week‐old male BALB/c nude mice (Vital River Laboratories, Beijing, China). Once the subcutaneous tumor reached 1 cm in diameter, it was minced into pieces (≈2 mm^3^) and then subcutaneously implanted into the flanks of 5‐week‐old male BALB/c nude mice. When the tumor volume reached ≈100 mm^3^ after implantation, the mice were randomly divided into three treatment groups for assessing the sensitivity of PDXs with different *IGFRIL* levels to IGF1R or mTOR inhibitors: 1) DMSO (intraperitoneal injection); 2) BMS‐754807 (25 mg kg^−1^ every 5 days, intraperitoneal injection; Cat. s1124, Selleck, USA); and 3) rapamycin (10 mg kg^−1^ every 5 days, intraperitoneal injection; Cat. s1039, Selleck, USA). The investigators were blinded to group medication during experiments and when assessing the outcomes. Tumor size was measured with a caliper once every seven days. After 30 days, all the mice were euthanized, and the primary tumors were excised and analyzed by H&E staining and IHC assays of Ki‐67, IGFBP3, p‐IGF1R, p‐AKT, and p‐mTOR. The tumor growth inhibition rate (TGI%) in drug‐treated mice relative to the controls was calculated as follows: 100% − (tumor volume of treated mice)/(average tumor volume of control mice).

The primary HCC cells (i.e., patient‐derived tumor cells [PDC]) were also isolated from HCC tissues of the above PDX models for drug sensitivity testing. Briefly, the HCC tissues from PDX models were minced using a razor blade and digested in collagenase digestion buffer at 37 °C for 1 h. Then, the cells were passed through 100‐µm and 40‐µm cell strainers and centrifuged at 1200 rpm for 5 min. Cell pellets were incubated in red blood cell (RBC) lysis buffer for 2 min and then re‐suspended in 6 mL medium and spun through 0.5 mL of serum layered on the bottom of the tube to remove the cellular debris. The contaminated human or mouse haematopoietic and endothelial cells were depleted using the biotin‐conjugated anti‐mouse CD45, CD31, and Ter119 antibodies and separated on a MACS LS column using anti‐biotin microbeads. The primary cells were cultured in hepatoma carcinoma cell medium (PreceDo Pharmaceuticals Co., Ltd., Hefei City, China) for mechanism and drug sensitivity tests as described in the “In vitro *assays in HCC cell lines*” section.

### Identification of the Dysregulated lncRNAs and Protein‐Coding Genes in HCC Based on Publicly Available Transcriptome Datasets

To investigate the dysregulated lncRNAs and protein‐coding genes in HCC tumor tissues relative to non‐tumor liver tissues, a transcriptome dataset from the Gene Expression Omnibus (GEO; Accession No. GSE54238)^[^ ^12^ ^]^ was used. This dataset contained a total of 56 tissue samples, including 10 normal livers (NL), 10 chronic hepatitis livers (IL), 10 cirrhotic livers (CL), 13 early HCC (eHCC), and 13 advanced HCC (aHCC) samples. All samples were subjected to transcriptome analyses using Arraystar human lncRNA microarray (V1‐100309, Rockville, USA), which contained ≈50 000 probes and systematically profiled the lncRNAs together with mRNAs on the same chip. Raw data were obtained, and expression data were extracted through NimbleScan software (Roche, Switzerland) by implementing robust multi‐array average (RMA). Probes with signal values ≥150 in at least 50% of samples were chosen for further analysis. The Global Rank‐invariant Set Normalization^[^ ^46^ ^]^ was employed to eliminate the intensity‐dependent bias of the transcript clusters dataset. Differentially expressed protein‐coding mRNAs (DEMs) and lncRNAs (DELs) between HCCs and the other three groups were identified using the limma package, and corrected *P*‐value < 0.05 and |log_2_(fold change)| ≥ 2 were considered to be statistically significant. Moreover, an independent lncRNA expression dataset containing 38 paired HCC tissues and non‐tumor liver tissues profiled by microarrays (GEO Accession No. GSE84006) from the previous study^[^ ^10^ ^]^ were also used for validation of the dysregulation of *IGFRIL*.

### Prediction of Protein‐Coding Potential of lncRNAs

The protein‐coding potential for lncRNAs was assessed using coding potential assessment tool (CAPT, http://lilab.research.bcm.edu/cpat),^[^ ^15^ ^]^ phylogenetic Codon Substitution Frequencies (PhyloCSF)^[^ ^19^ ^]^ and coding potential calculator (CPC, http://cpc.cbi.pku.edu.cn/).^[^ ^14^ ^]^ PhyloCSF examines evolutionary signatures characteristic of alignments of conserved coding regions, and the PhyloCSF scores based on 29‐mammal genome alignment were obtained using the conservation track from the UCSC genome browser for hg19.^[^ ^47^ ^]^ The ORFfinder, CAPT, and CPC were run using their online tools. The *ACTB* and *GAPDH* mRNAs were used as positive controls, while the well‐known lncRNAs *HOTAIR* and *LINK‐A* were used as negative controls.

### In silico Prediction of RNA Secondary Structure

The prediction of secondary structure of lncRNA *IGFRIL* was performed by RNAfold WebServer (http://rna.tbi.univie.ac.at/cgi‐bin/RNAfold.cgi), which was based on the minimum free energy (MFE) and partition function as described previously.^[^ ^48^ ^]^

### RNA‐Seq and Pathway Enrichment Analyses

The total RNAs were extracted from HepG2 cells, which were transfected with si‐scramble, si‐*IGFRIL*, or si‐*PTBP1*. RNA sequencing (RNA‐seq) libraries were constructed using the VAHTS mRNA‐seq v2 Library Prep Kit for Illumina (Vazyme, China) according to the manufacturer's protocol. The library was quality‐checked and sequenced using the NovaSeq 6000 sequencer (Illumina, USA). The quality of raw sequencing reads was evaluated using FastQC. Adaptor sequences and low‐quality score bases were trimmed using Trim Galore (v.0.6.7; https://github.com/FelixKrueger/TrimGalore). These reads were then mapped to the human genome reference GRCh38 from Ensembl release 105 using Bowtie2 (v.2.2.5).^[^ ^49^ ^]^ The fragments per kilobase of exon per million mapped reads values and gene count values were computed using RSEM (v.1.2.8)^[^ ^50^ ^]^ and the DEGs were analyzed using the DESeq2 (v.1.37) R package.^[^ ^51^ ^]^ Genes with *P*‐value < 0.05 and fold‐change > 1.2 were considered to be statistically significant. Differentially alternative splicing events (DASEs) were identified by SUPPA2.^[^ ^52^ ^]^ Events with *P* < 0.05 and delta percent spliced‐in (Δ*PSI*) value > 0.05 were considered to be statistically significant. The significance of the overlaps for the DEGs or DASEs between the *IGFRIL*‐ and *PTBP1*‐knockdown experiments was computed by a hypergeometric test. The functional enrichment analysis of the DEGs was performed using the clusterProfiler (v.4.2.2) R package,^[^ ^53^ ^]^ the gene ontology (GO) annotations and canonical pathways (KEGG and Reactome) gene sets were obtained from MSigDB (v2022.1.Hs; www.broadinstitute.org/gsea/msigdb). Gene set enrichment analysis (GSEA) was performed based on the paired groups (knockdown vs control), with the genes ranked by their signal‐to‐noise ratios. Then, the significant gene sets from MsigDB were identified by the weighted Kolmogorov–Smirnov test. Gene sets were permuted 1000 times to compute the false discovery rates (FDRs).

### High‐Throughput Sequencing of RNA Isolated by Cross‐Linking Immunoprecipitation (CLIP) Assays

The *IGFRIL*‐knocked‐down and control HepG2 cells were cross‐linked by UV light (254 nm) to maintain covalent binding of RBPs to their cognate RNAs. Then, the PTBP1 and cross‐linked RNAs were immunoprecipitated using antibodies against PTBP1 (32‐4800; Thermo Fisher, USA) and digested with Micrococcal nuclease (EN0181; Thermo Fisher Scientific, USA). CLIP assays by IgG antibody (ab172730; Abcam, USA) were used as a negative control. An IR800‐biotin adapter was ligated to the 3′ end of the RNA fragment. Then, the PTBP1/RNA complexes were separated by SDS‐PAGE gel and transferred to the nitrocellulose membrane (HATF00010; Millipore, USA). These RNA and protein complexes were digested by proteinase K (9034; TaKaRa, Japan). RNAs were isolated using saturated phenol (AM9712; Ambion, USA), ligated with adaptors, and converted to cDNAs by the SuperScript III First‐Strand Kit (18080‐051; Invitrogen, USA). The cDNAs were amplified by PCR to construct the corresponding libraries and were then sequenced on the Illumina HiSeq 2500 platform.

The CLIP‐seq data were analyzed as previously reported.^[^ ^54^ ^]^ Reads were first trimmed of adaptor sequences by Trimmomatic (version 0.36). Then, clean reads were mapped to the hg38 reference genome by Bowtie 2 (version 2.1.0) with parameters “‐p 10 ‐L 15 ‐N 1 ‐D 50 ‐R 50 –phred33 –qc‐filter –very‐sensitive –end‐to‐end”. CLIP‐seq peaks were identified by Piranha (version 1.2.1) with the following parameters: “‐s ‐b 20 ‐d Zero Truncated Negative Binomial ‐p 0.05”. CLIP‐seq signals were calculated as aggregated read counts normalized to the length of the regions and the total mappable read counts, resulting in reads per million mapped reads (RPM) as the measurement unit.

### Statistical Analyses

All results were confirmed in at least three independent experiments, and data were represented as mean ± standard error of mean (SEM) or standard deviation (SD). For those datasets that fulfilled Pearson normality test criteria, statistical significance of differences between 2 groups was evaluated by 2‐sided unpaired Student's *t‐*test, and statistical significance of differences among multiple groups was analyzed by 1‐way ANOVA; otherwise, non‐parametric Wilcoxon signed‐rank test was chosen. Fisher's exact test or *χ* ^2^ test was used for the analyses of contingency tables depending on the sample sizes. Kaplan–Meier method was used to estimate the survival rate, and the difference between survival curves of the two groups was assessed by the log‐rank test. The hazard ratio (HR) and 95% confidence interval (CI) were calculated using the univariate Cox proportional hazards regression analysis. Differences were considered to be statistically significant at *P* < 0.05. All the statistical analyses, except where otherwise noted, were performed using R version 3.3.0 software (www.rproject.org) or GraphPad Prism (version 9).

## Conflict of Interest

The authors declare no conflict of interest.

## Author Contributions

J.Z., C.G., H.L., Y.C., and L.Y. contributed equally to this work. G.Z. was the overall principal investigator who conceived the study and obtained financial supports. G.Z. and P.C. designed and supervised the project. PC collected omics data and interpreted the results. J.Z., H.L., L.Y., and C.G. performed the functional assays. Q.J., L.F., X.W., X.L., H.C., G.Z., X.Z., Q.Z., Q.Z., Y.C., G.Q., and A.Y. helped to perform the functional assays. C.G., M.C., and X.C. helped collected omics data. Y.X. and X.C. helped to perform the CLIP assays. H.W., M.Z., A.S., and Q.S. were responsible for recruitment of HCC patients, and collection of their clinical information and biological specimens. H.J. and M.Y. were responsible for recruitment of CRC and GC patients, respectively, and collection of their clinical information and biological specimens. Y.L. and Y.W. helped to collect the biological specimens and clinical information of HCC patients for PDX studies. P.C. and J.Z. conducted samples selection and data management, performed the statistical analyses, and interpreted the results. G.Z., P.C., and H.L. drafted the manuscript. All authors read and approved the final manuscript.

## Supporting information



Supporting Information

Supplemental Table 1

## Data Availability

The accession number in Gene Expression Omnibus (GEO) for the gene expression profile data of HepG2 cells upon *IGFRIL* or *PTBP1* knockdown is GSE232354; the accession number for the CLIP‐seq data of HepG2 cells upon *IGFRIL* knockdown in GEO is GSE232353.
