# Supplementary material for: Long Non‐Coding RNA IGFRIL Couples with PTBP1 to Destabilize IGFBP3 mRNA to Promote the IGF1R‐AKT‐mTOR Axis and Hepatocellular Carcinoma
Source: Adv Sci (Weinh). 2025 Jul 21;12(39):e07676. doi: 10.1002/advs.202507676 (PMC12533367; doi:10.1002/advs.202507676)
Supplement: Supplementary file 1 — Supporting Information [file ADVS-12-e07676-s002.docx]

**Long non-coding RNA *IGFRIL* couples with PTBP1 to destabilize *IGFBP3* mRNA to promote the IGF1R-AKT-mTOR axis and hepatocellular carcinoma**

**Short title:** *IGFRIL* promotes HCC by activating the IGF1R signaling

Jing Zhang^1,2,#^, Chengming Gao^1,#^, Haibei Li^1,3,#^, Yuying Chen^1,4,#^, Liting Yang^1,5,#^, Qian Jin^1,6^, Lan Feng^1^, Xinyi Liu^1^, Hongxia Chen^1^, Rong Ye^7^, Yuanchao Xue^7^, Huiyun Wang^8^, Musheng Zeng^8^, Ming Yang^9^, Huaiqiang Ju^8^, Chengzhi Gao^1^, Guangming Zhou^1^, Qi Zhang^4^, Qian Zhang^10,11^, Geng Qin^1^, Yuanfeng Li^1^, Yahui Wang^1^, Aiqing Yang^1^, Anfeng Si^12^, Qingfeng Song^13^, Fuchu He^14^, Pengbo Cao^1,*^, Gangqiao Zhou^1,2,4,10,15,*^

^1^State Key Laboratory of Medical Proteomics, National Center for Protein Sciences at Beijing, Beijing Institute of Radiation Medicine, Beijing, P.R. China;

^2^Institute of Life Science and Green Development, College of Life Sciences, Hebei University, Baoding City, Hebei Province, P.R. China;

^3^Military Medical Sciences Academy, Tianjin, P.R. China;

^4^University of South China, Hengyang City, Hunan Province, P.R. China;

^5^Department of Neurosurgery, Xiangya Hospital, Central South University, Changsha City, Hunan Province, P.R. China;

^6^Jiangsu Key Laboratory of Biological Cancer Therapy, Xuzhou Medical College, Xuzhou City, Jiangsu Province, P.R. China;

^7^Key Laboratory of RNA Biology, Institute of Biophysics, Chinese Academy of Sciences, Beijing, P.R. China;

^8^State Key Laboratory of Oncology in South China, Collaborative Innovation Center for Cancer Medicine, Sun Yat-Sen University Cancer Center, Guangzhou City, Guangdong Province, P.R. China;

^9^Cancer Center at Shandong University, Jinan City, Shandong Province, P.R. China;

^10^Medical College of Guizhou University, Guiyang City, Guizhou Province, P.R. China;

^11^West China Hospital of Sichuan University, Chengdu City, Sichuan Province, P.R. China;

^12^Affiliated Jinling Hospital, Medical School of Nanjing University, Nanjing City, Jiangsu Province, P.R. China;

^13^Affiliated Cancer Hospital of Guangxi Medical University, Nanning City, Guangxi Zhuang Autonomous Region, P.R. China;

^14^State Key Laboratory of Medical Proteomics, National Center for Protein Sciences at Beijing, Beijing Institute of Lifeomics, Beijing, P.R. China;

^15^Collaborative Innovation Center for Personalized Cancer Medicine, Center for Global Health, School of Public Health, Nanjing Medical University, Nanjing City, Jiangsu Province, P.R. China;

^#^These authors contributed equally to this work.

^*^**Correspondence and address requesting for reprints to:**

Dr. Gangqiao Zhou, State Key Laboratory of Medical Proteomics, National Center for Protein Sciences at Beijing, Beijing Institute of Radiation Medicine, 27 Taiping Road, Beijing 100850, P.R. China. E-mail: zhougq114@126.com; Phone & fax: (86)-010-66931201.

OR

Dr. Pengbo Cao, State Key Laboratory of Medical Proteomics, National Center for Protein Sciences at Beijing, Beijing Institute of Radiation Medicine, 27 Taiping Road, Beijing 100850, P.R. China. E-mail: birchcpb@163.com; Phone: (86)-010-61777099.

**This file includes:**

**Figures S1 - S11:**

Figure S1. The characterization of the HCC-associated lncRNA *IGFRIL*.

Figure S2. *IGFRIL* plays oncogenic roles in HCC *in vitro* and *in vivo*.

Figure S3. PTBP1 plays an oncogenic role in HCC cells.

Figure S4. *IGFRIL* plays oncogenic roles dependent on PTBP1 in HCC cells.

Figure S5. *IGFRIL* couples with PTBP1 to enhance the activation of AKT-mTOR signaling in HCC cells.

Figure S6. *IGFRIL* functions dependent on the AKT-mTOR signaling in HCC cells.

Figure S7. *IGFRIL* couples with PTBP1 to reduce *IGFBP3* mRNA levels in HCC cells.

Figure S8. *IGFRIL* couples with PTBP1 to destabilize *IGFBP3* mRNA in HCC cells.

Figure S9. IGFBP3 reduces the activities of the AKT-mTOR signaling and plays a tumor-suppressive role in HCC cells.

Figure S10. *IGFRIL* couples with PTBP1 to reduce *IGFBP3* expression to activate the IGF1R-AKT-mTOR signaling and promote HCC progression.

Figure S11. Upregulation of *IGFRIL* sensitizes HCC cells to IGF1R and mTOR inhibitors.

**Tables S1 - S8** (shown in Supplementary Excel file)**:**

Table S1. The differentially expressed mRNAs and lncRNAs between HCC tissues and non-tumor liver tissues.

Table S2. Major demographic and clinicopathological characteristics of cancer patients in this study.

Table S3. Correlations between the *IGFRIL* expression levels and clinicopathologic characteristics in patients from the three HCC cohorts.

Table S4. Univariate and multivariate analyses of factors associated with OS and DFS in patients from the three HCC cohorts.

Table S5. The proteins interacted with *IGFRIL* identified by RNA pull-down and mass spectrometry.

Table S6. The overlapped differentially alternative splicing events in HepG2 cells with *IGFRIL* knockdown and *PTBP1* knockdown.

Table S7. The overlapped differentially expressed genes in HepG2 cells with *IGFRIL* knockdown and *PTBP1* knockdown.

Table S8. Probes, primers and siRNAs used in this study.


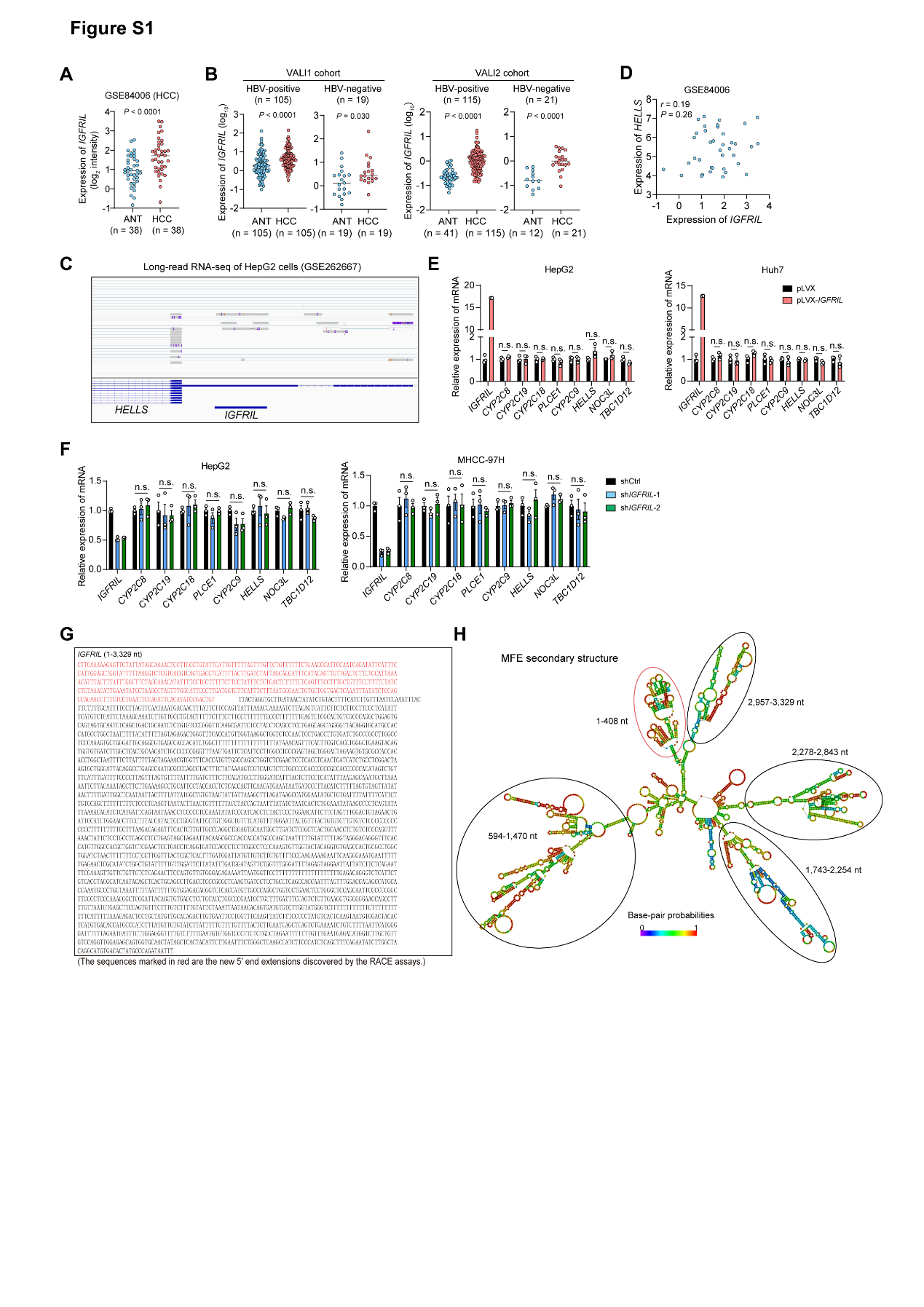


**Figure S1. The characterization of the HCC-associated lncRNA *IGFRIL*.**

(A) *IGFRIL* expression levels in tumors and adjacent non-tumor (ANT) liver tissues of the HCC patients from the GSE84006 dataset. (B) *IGFRIL* expression levels in tumors and adjacent non-tumor (ANT) liver tissues of HBV-positive or HBV-negative HCC patients from the validation cohorts (VALI1 and VALI2). *P* values were calculated using Student’s *t* test. (C) Regional view of RNA reads mapped to the *IGFRIL* locus by long-read RNA sequencing assays in HepG2 cells (GSE262667). (D) The correlation between *IGFRIL* levels and *HELLS* mRNA levels based on GSE84006 dataset. The *r* value and *P* value are calculated by Spearman’s correlation analysis. (E) and (F) Relative mRNA levels of 8 genes (including *HELLS*, *PLCE1*, *NOC3L*, *TBC1D12*, *CYP2C18*, *CYP2C19*, *CYP2C9* and *CYP2C8*) within the 1 megabase (Mb) region centered on the *IGFRIL* locus in HepG2, MHCC97H and Huh7 cells upon overexpression or knockdown of *IGFRIL*. (G) The nucleotide sequence of full-length *IGFRIL*. The sequences marked in red are the new 5' end extensions discovered by the rapid amplification of cDNA ends (RACE) assays. (H) Graphic illustration of the predicted secondary structure of *IGFRIL* using RNAfold based on minimum free energy (MFE) algorithm. The results are expressed as mean ± standard error of mean (SEM) of three independent experiments. Statistical analysis was performed using the 2-sided, unpaired Student’s *t* test (A, B and E) or 1-way ANOVA (F). n.s., not significant.


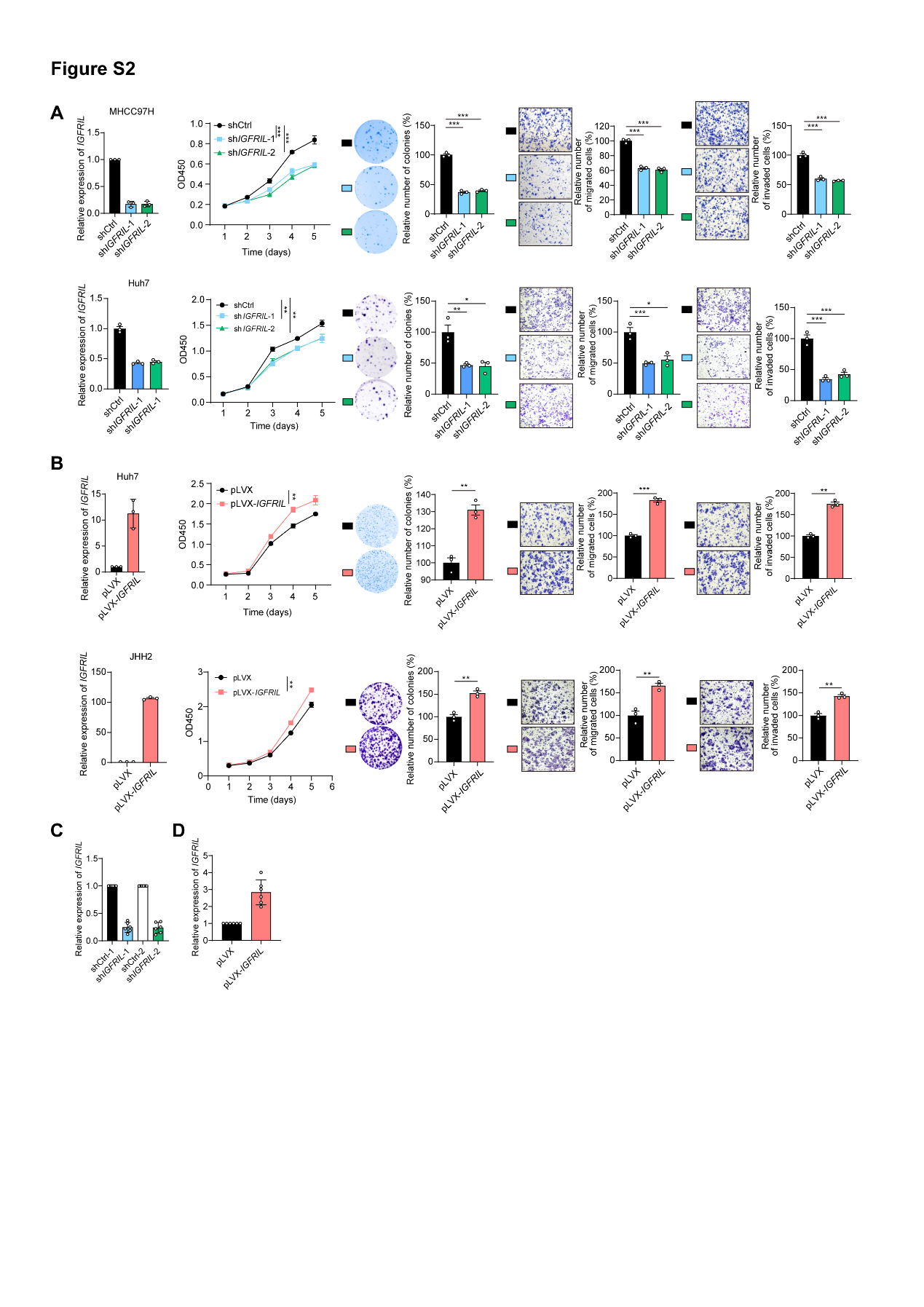


**Figure S2. *IGFRIL* plays oncogenic roles in HCC *in vitro* and *in vivo*.**

(A) The effects of *IGFRIL* knockdown on the cells proliferation, plate colony formation, migration and invasion in MHCC97H (top) and Huh7 (bottom) cells. (B) The effects of *IGFRIL* overexpression on the cells proliferation, plate colony formation, migration and invasion in Huh7 (top) and JHH2 (bottom) cells. (C) Confirmation of efficiency of *IGFRIL* knockdown by qRT-PCR assays for *IGFRIL* levels in subcutaneous tumor tissues in Figure 2G. (D) Confirmation of *IGFRIL* overexpression efficiency by qRT-PCR assays for *IGFRIL* levels in subcutaneous tumor tissues in Figure 2I. The results are expressed as mean ± standard error of mean (SEM) of three or more independent experiments. Statistical analysis was performed using the 2-sided, 1-way ANOVA (A) or unpaired Student’s *t* test (B). ^*^, *P* < 0.05; ^**^, *P* < 0.01; ^***^, *P* < 0.001.


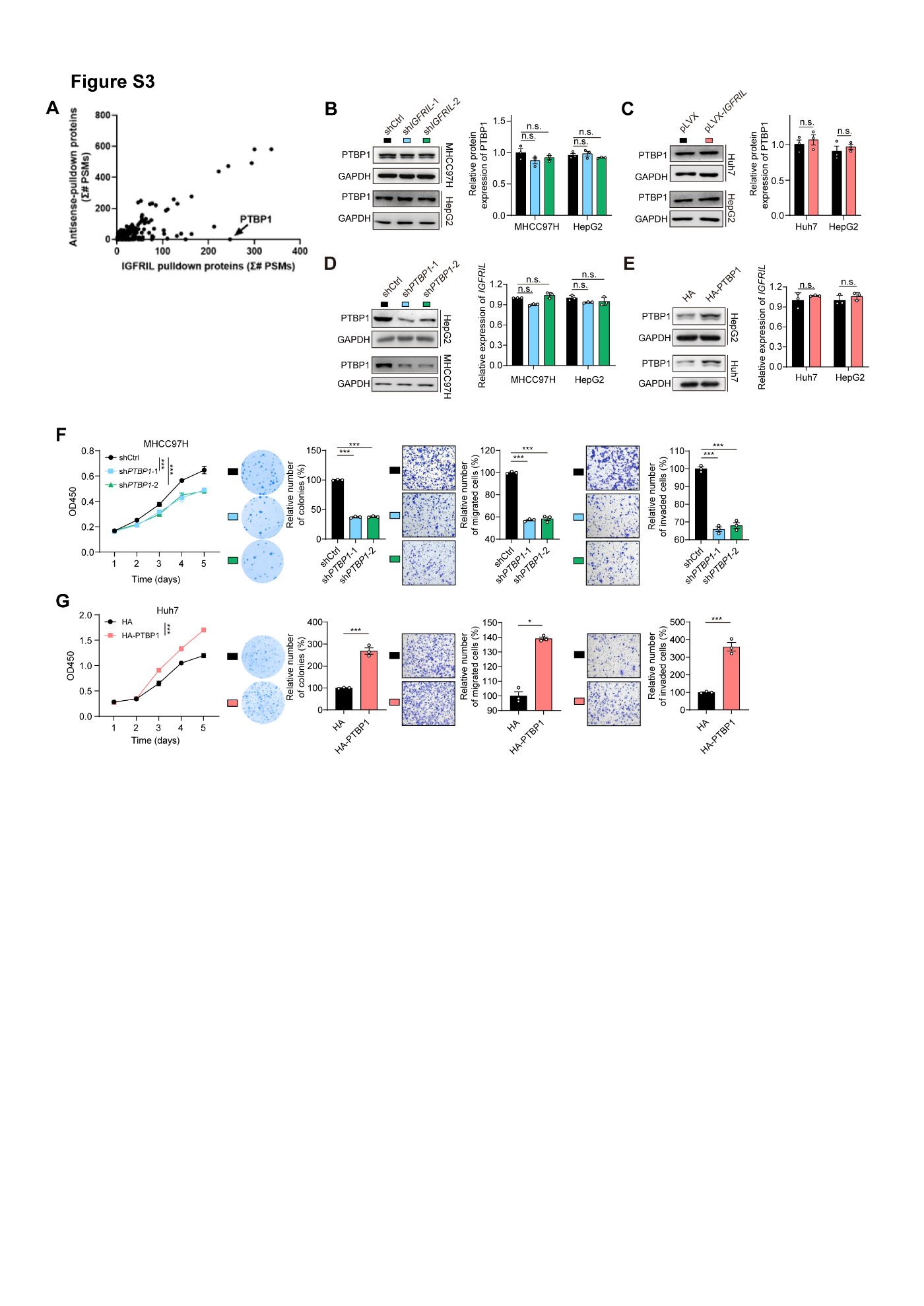


**Figure S3.** **PTBP1 plays an oncogenic role in HCC cells.**

(A) Comparison of the abundance of *IGFRIL*-associated proteins and antisense-associated proteins after RNA pull-down in HepG2 cells. The pulled-down proteins were subjected to tandem mass spectrum analyses. PTBP1 with peptide-spectrum matches (PSM) > 250 was specifically pulled-down by *IGFRIL*. (B) and (C) The effects of *IGFRIL* knockdown (B) or overexpression (C) on expression of PTBP1 protein in HepG2, MHCC97H or Huh7 cells, respectively, by immunoblotting (IB) assays. (D) and (E) The effects of *PTBP1* knockdown (D) or overexpression (E) on expression of *IGFRIL* in HepG2, MHCC97H or Huh7 cells, respectively, by real-time quantitative reverse transcription polymerase chain reaction (qRT-PCR) assays. (F) and (G) The effects of *PTBP1* knockdown in MHCC97H cells (F) or PTBP1 overexpression in Huh7 cells (G) on cells proliferation, plate colony formation, migration and invasion. The results are expressed as mean ± standard error of mean (SEM) of three or more independent experiments. Statistical analysis was performed using the 2-sided, 1-way ANOVA (B, D and F) or unpaired Student’s *t* test (C, E and G). ^*^, *P* < 0.05; ^***^, *P* < 0.001; n.s., not significant.


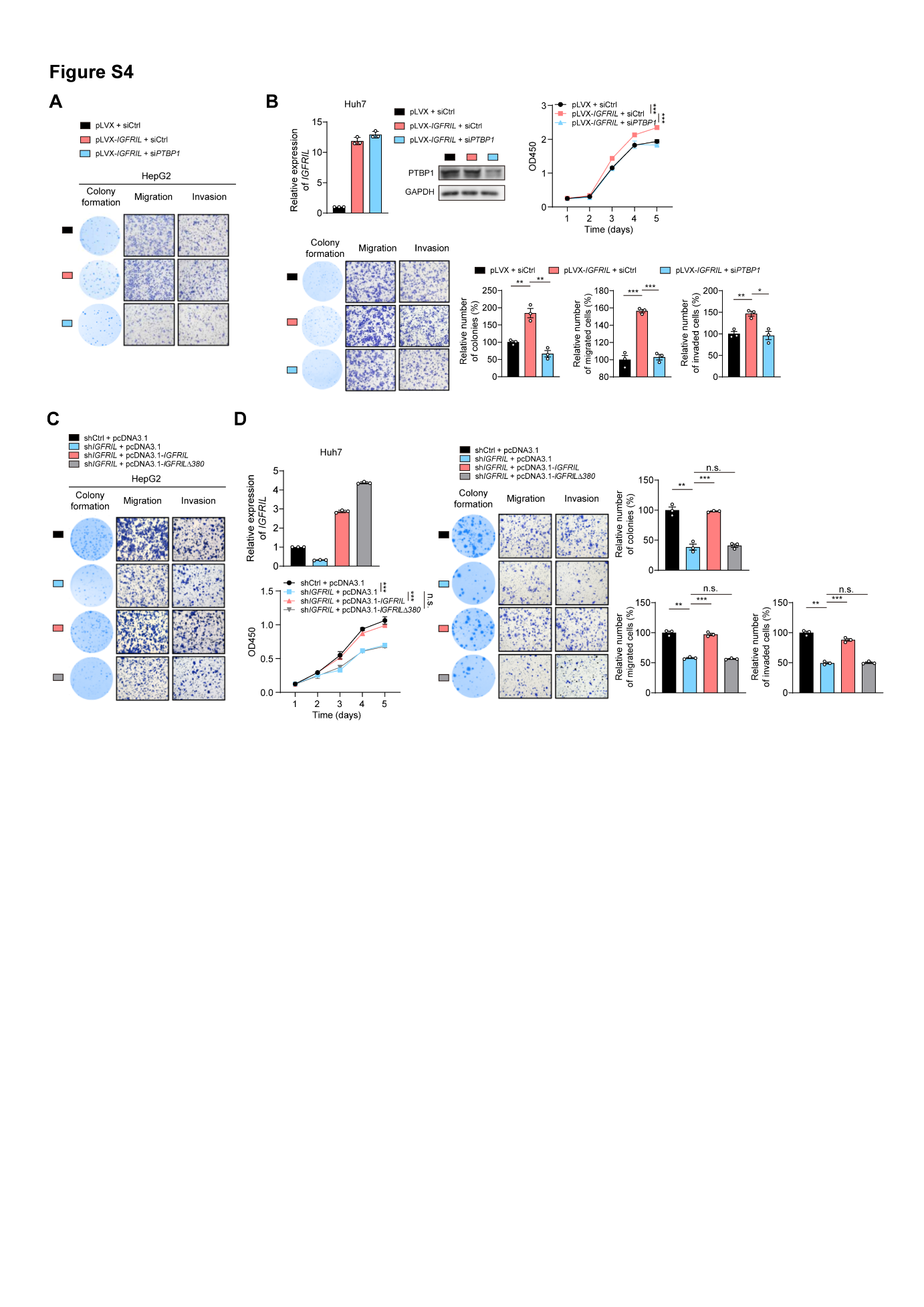


**Figure S4. *IGFRIL* plays oncogenic roles dependent on PTBP1 in HCC cells.**

(A) The effects of *PTBP1* knockdown on the increased cell plate colony formation, migration and invasion by *IGFRIL* overexpression in HepG2 cells. (B) The effects of *PTBP1* knockdown on the increased cell proliferation, plate colony formation, migration and invasion by *IGFRIL* overexpression in Huh7 cells. (C) Re-expression of full-length *IGFRIL*, but not mutant *IGFRIL△380*, restores the decreased plate colony formation, migration and invasion by *IGFRIL* knockdown in HepG2 cells. (D) Re-expression of full-length *IGFRIL*, but not mutant *IGFRIL△380*, restores the decreased proliferation, plate colony formation, migration and invasion by *IGFRIL* knockdown in Huh7 cells. The results are expressed as mean ± standard error of mean (SEM) of three or more independent experiments. Statistical analysis was performed using the 2-sided, 1-way ANOVA (B and D). ^*^, *P* < 0.05; ^**^, *P* < 0.01; ^***^, *P* < 0.001; n.s., not significant.


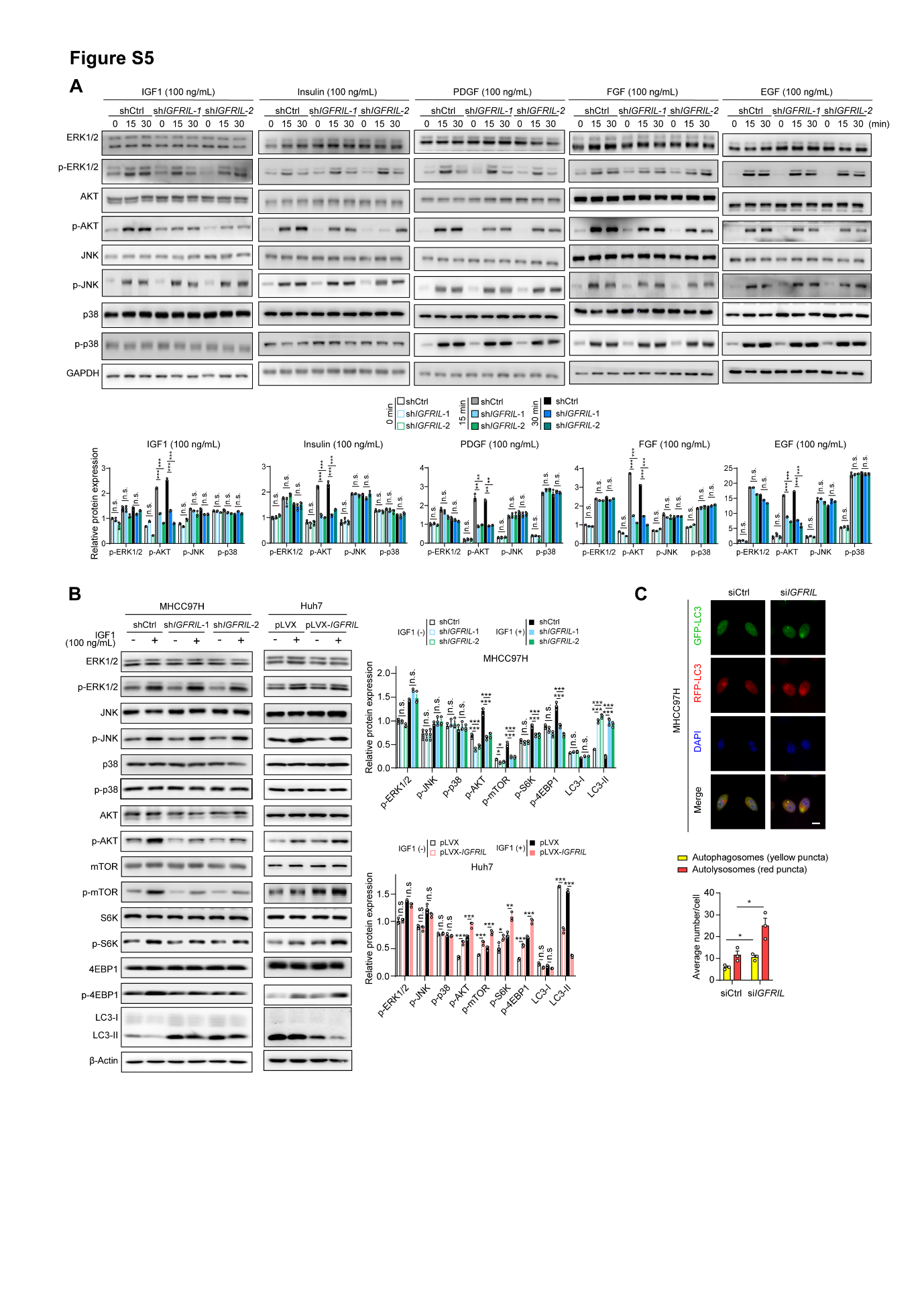


**Figure S5. *IGFRIL* couples with PTBP1 to enhance the activation of AKT-mTOR signaling in HCC cells.**

(A) The effects of *IGFRIL* knockdown on the activation of the key members downstream of the receptor tyrosine kinases (RTK) signaling, including ERK1/2, JNK, p38 and AKT, in HepG2 cells without or with the treatment of IGF1 (100 ng/mL), insulin (100 ng/mL), PDGF (100 ng/mL), FGF2 (100 ng/mL) or EGF (100 ng/mL) for 15 or 30 minutes. (B) The effects of *IGFRIL* knockdown in MHCC97H cells (left) or *IGFRIL* overexpression in Huh7 cells (right) on the activities of ERK1/2, JNK, p38 and AKT and the downstream mTOR signaling members, without or with IGF1 treatment for 30 min. **(C)** Knockdown of *IGFRIL* accelerates the autophagic flux in MHCC97H cells. MHCC97H cells stably expressing mRFP-GFP-LC3 were transfected with siCtrl or si*IGFRIL*, followed by staining with DAPI. Red puncta represent the autophagosomes; and yellow puncta in merged picture represent the autolysosomes. Scale bars, 40 µm. The results are expressed as mean ± standard error of mean (SEM) of three or more independent experiments. Statistical analysis was performed using the 2-sided, 1-way ANOVA (A and B). ^*^, *P* < 0.05; ^**^, *P* < 0.01; ^***^, *P* < 0.001; n.s., not significant.


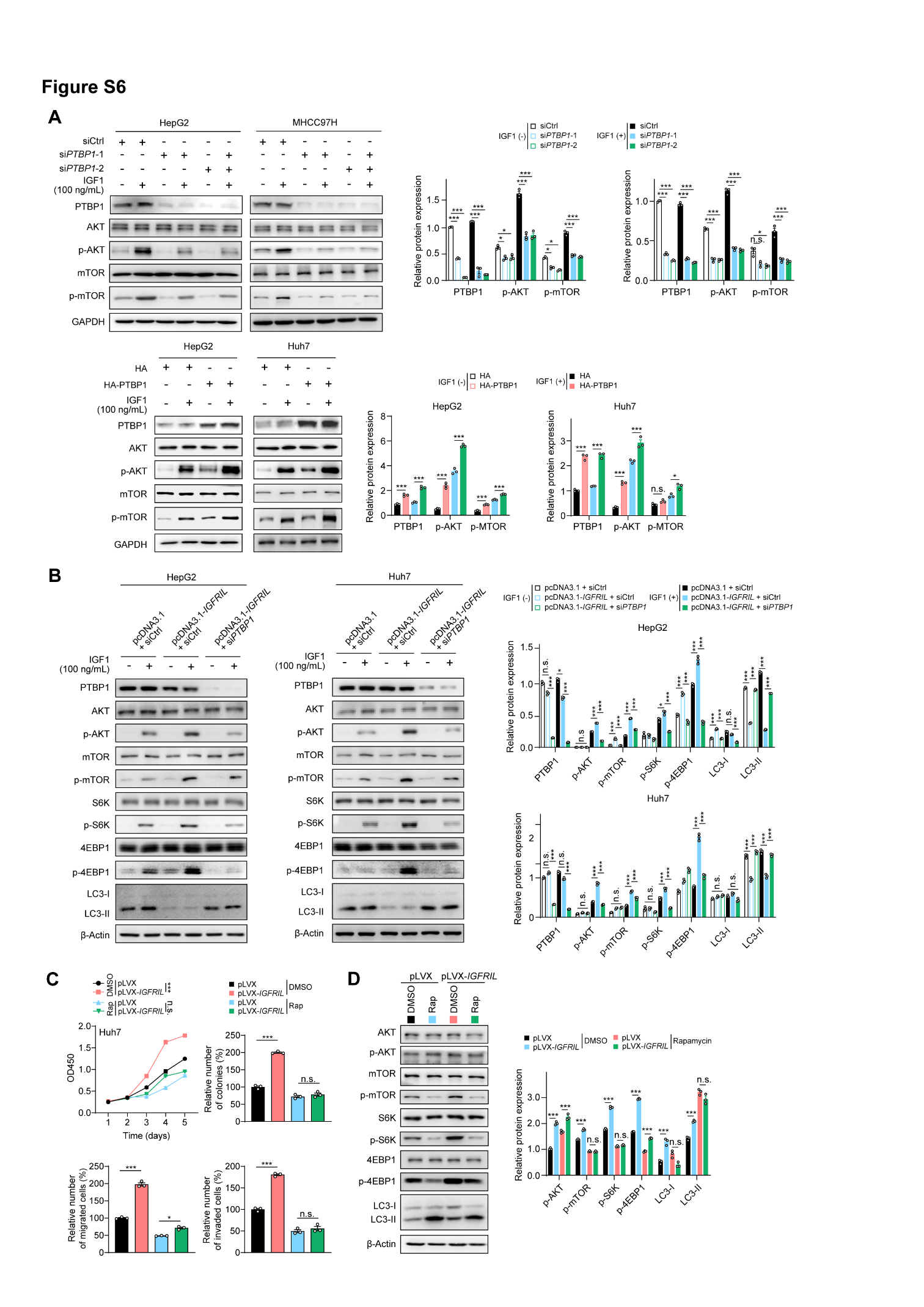


**Figure S6. *IGFRIL* functions dependent on the AKT-mTOR signaling in HCC cells.**

(A) The effects of *PTBP1* knockdown in HepG2 or MHCC97H cells (top), and *PTBP1* overexpression in HepG2 or Huh7 cells (bottom), on the activities of the AKT-mTOR singling without or with IGF1 treatment (100 ng/mL). (B) The effects of *PTBP1* knockdown on the increased activities of the AKT-mTOR signaling induced by *IGFRIL* overexpression in HepG2 (left) and Huh7 cells (right). (C) and (D) The effects of mTOR inhibitor rapamycin (10 μM) on the increased proliferation, plate colony formation, migration and invasion (C), and the activities of the AKT-mTOR signaling (D) induced by *IGFRIL* overexpression in Huh7 cells. The results are expressed as mean ± standard error of mean (SEM) of three or more independent experiments. Statistical analysis was performed using the 2-sided, 1-way ANOVA. ^*^, *P* < 0.05; ^***^, *P* < 0.001; n.s., not significant.


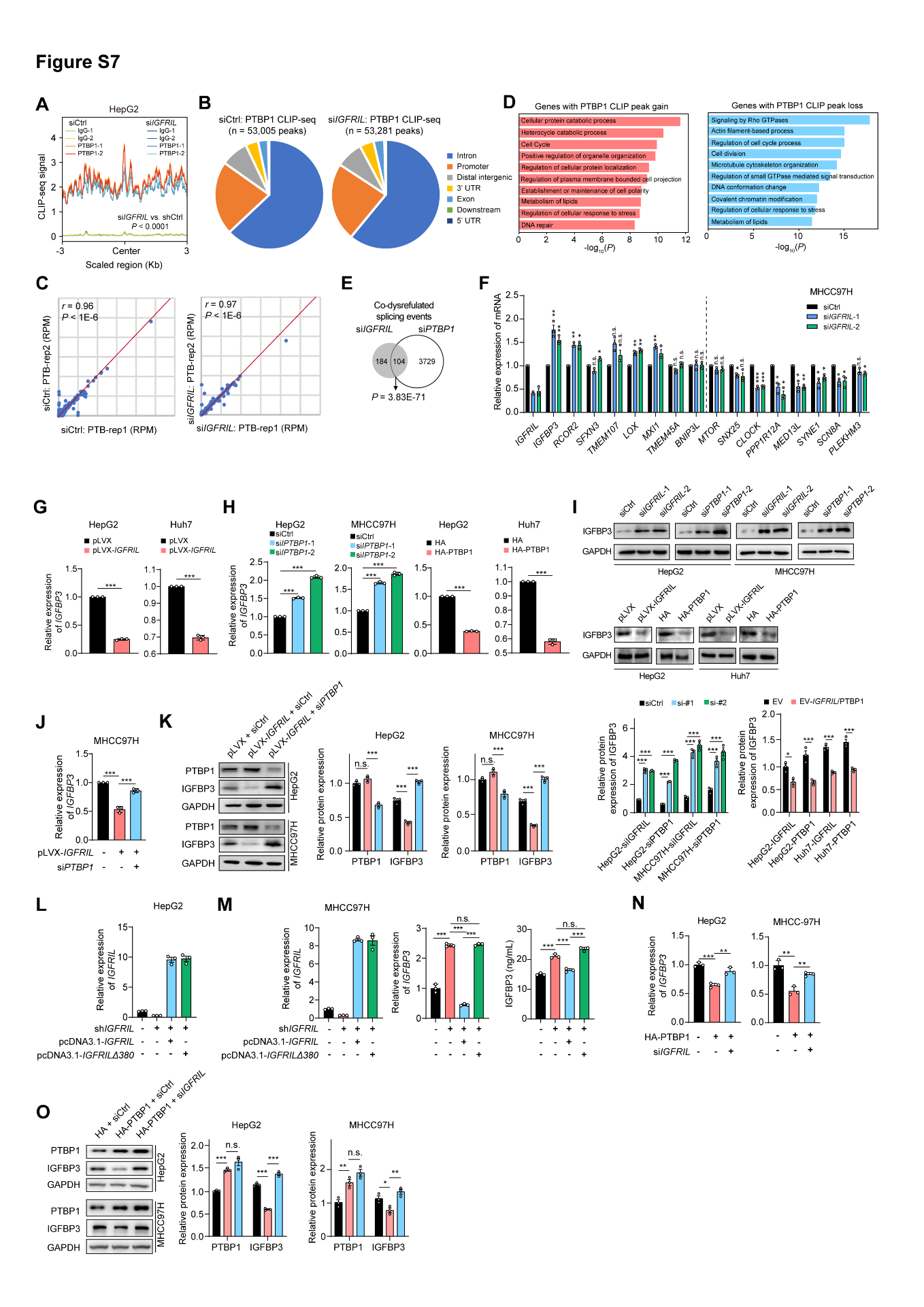


**Figure S7. *IGFRIL* couples with PTBP1 to reduce *IGFBP3* mRNA levels in HCC cells.**

(A) The distribution of PTBP1 binding sites in ~6 kilobase pairs (Kb) region surrounding the center of the binding sites in *IGFRIL*-knocked-down and control HepG2 cells. The *x*-axis denotes the position from the center of the binding sites, and *y*-axis shows the signal density (mean density of the reads). The cross-linked immunoprecipitation (CLIP) assays by IgG antibody were used as negative control. Each group has two replicates. (B) Genome-wide distribution of PTBP1 binding sites. (C) The correlation of the PTBP1 binding peaks intensities determined by CLIP sequencing (CLIP-seq) between replicates. The significance was assessed by Pearson’s correlation analysis. (D) KEGG and GO enrichment analyses of genes with PTBP1 CLIP-seq peaks gain (left), or loss (right) in HepG2 cells under *IGFRIL* knockdown. (E) Co-dysregulated alternative splicing events by *IGFRIL* knockdown and *PTBP1* knockdown in HepG2 cells. The significance of overlaps was computed by hypergeometric test. (F) The effects of *IGFRIL* knockdown on the mRNA levels of the 16 candidate genes in MHCC97H cells by real-time quantitative reverse transcription polymerase chain reaction (qRT-PCR) assays. (G) The effects of *IGFRIL* overexpression on *IGFBP3* mRNA levels in HepG2 and Huh7 cells. (H) The effects of *PTBP1* knockdown or overexpression on *IGFBP3* mRNA levels in HepG2, MHCC97H and Huh7 cells. (I) The effects of knockdown of *IGFRIL* or *PTBP1*, or overexpression of *IGFRIL* or PTBP1 on IGFBP3 protein levels determined by immunoblotting (IB) assays in HepG2, MHCC97H and Huh7 cells. (J) The effects of *PTBP1* knockdown on the decreased *IGFBP3* mRNA levels by *IGFRIL* overexpression in MHCC97H cells. (K) The effects of *PTBP1* knockdown on the decreased IGFBP3 protein levels by *IGFRIL* overexpression in HepG2 and MHCC97H cells. (L) Conformation the efficiencies of knockdown or re-expression of *IGFRIL* in HepG2 cells by qRT-PCR assays. (M) Re-expression of full-length *IGFRIL*, but not its mutant *IGFRIL△380*, abolishes the increased levels of mRNA and secreted protein of IGFBP3 by *IGFRIL* knockdown in MHCC97H cells. (N) The effects of *IGFRIL* knockdown on the decreased *IGFBP3* mRNA levels by PTBP1 overexpression in HepG2 and MHCC97H cells. (O)The effects of *IGFRIL* knockdown on the decreased IGFBP3 protein levels by PTBP1 overexpression in HepG2 and MHCC97H cells. The results are expressed as mean ± standard error of mean (SEM) of three or more independent experiments. Statistical analysis was performed using the 2-sided, 1-way ANOVA (F and H-O) or unpaired Student’s *t* test (G). ^*^, *P* < 0.05; ^**^, *P* < 0.01; ^***^, *P* < 0.001; n.s., not significant.


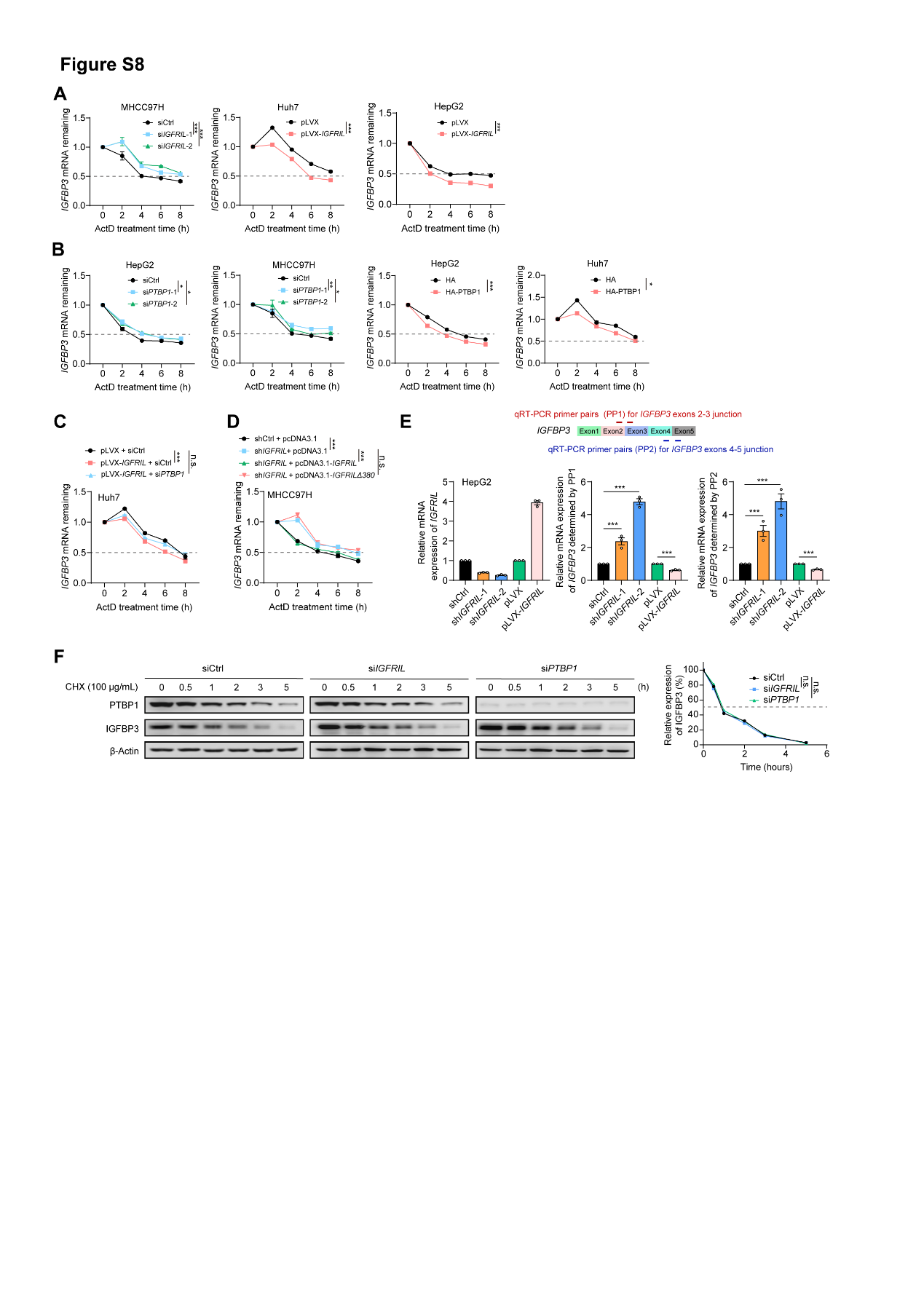


**Figure S8. *IGFRIL* couples with PTBP1 to destabilize *IGFBP3* mRNA in HCC cells.**

(A) The stability of *IGFBP3* mRNA in *IGFRIL*-knocked-down MHCC97H cells (left), or *IGFRIL*-overexpressed Huh7 and HepG2 cells (middle and right) after treatment with actinomycin D (ActD; 5 µg/mL) for indicated times. (B) The stability of *IGFBP3* mRNA in *PTBP1*-knocked-down HepG2 and MHCC97H cells (left), or PTBP1-overexpressed HepG2 and Huh7 cells (right) after treatment with ActD (5 µg/mL) for indicated times. (C) The effects of *PTBP1* knockdown on the decreased *IGFBP3* mRNA levels by *IGFRIL* overexpression in Huh7 cells. (D) Re-expression of full-length *IGFRIL*, but not the mutant *IGFRIL△380*, re-shortens the increased stability of *IGFBP3* mRNA by *IGFRIL* knockdown in MHCC97H cells. (E) The effect of *IGFRIL* on the alternative splicing of *IGFBP3*. The qRT-PCR assays based on the exon junction-specific (exon 2 - 3 junction and exon 4 - 5 junction) primers were used to determine the effects of knockdown or overexpression of *IGFRIL* on the mRNA levels of *IGFBP3* with specific exon junctions in HepG2 cells. PP1, primer pair1; PP2, primer pair 2. (F) The stability of IGFBP3 protein after treatment with cycloheximide (CHX, 100 μg/mL) for indicated times in HepG2 cells under knockdown of *IGFRIL* or *PTBP1*. The results are expressed as mean ± standard error of mean (SEM) of three independent experiments. Statistical analysis was performed using the 2-sided, repeated measures 1-way ANOVA (A-D and F) or 1-way ANOVA (e). ^*^, *P* < 0.05; ^**^, *P* < 0.01; ^***^, *P* < 0.001.


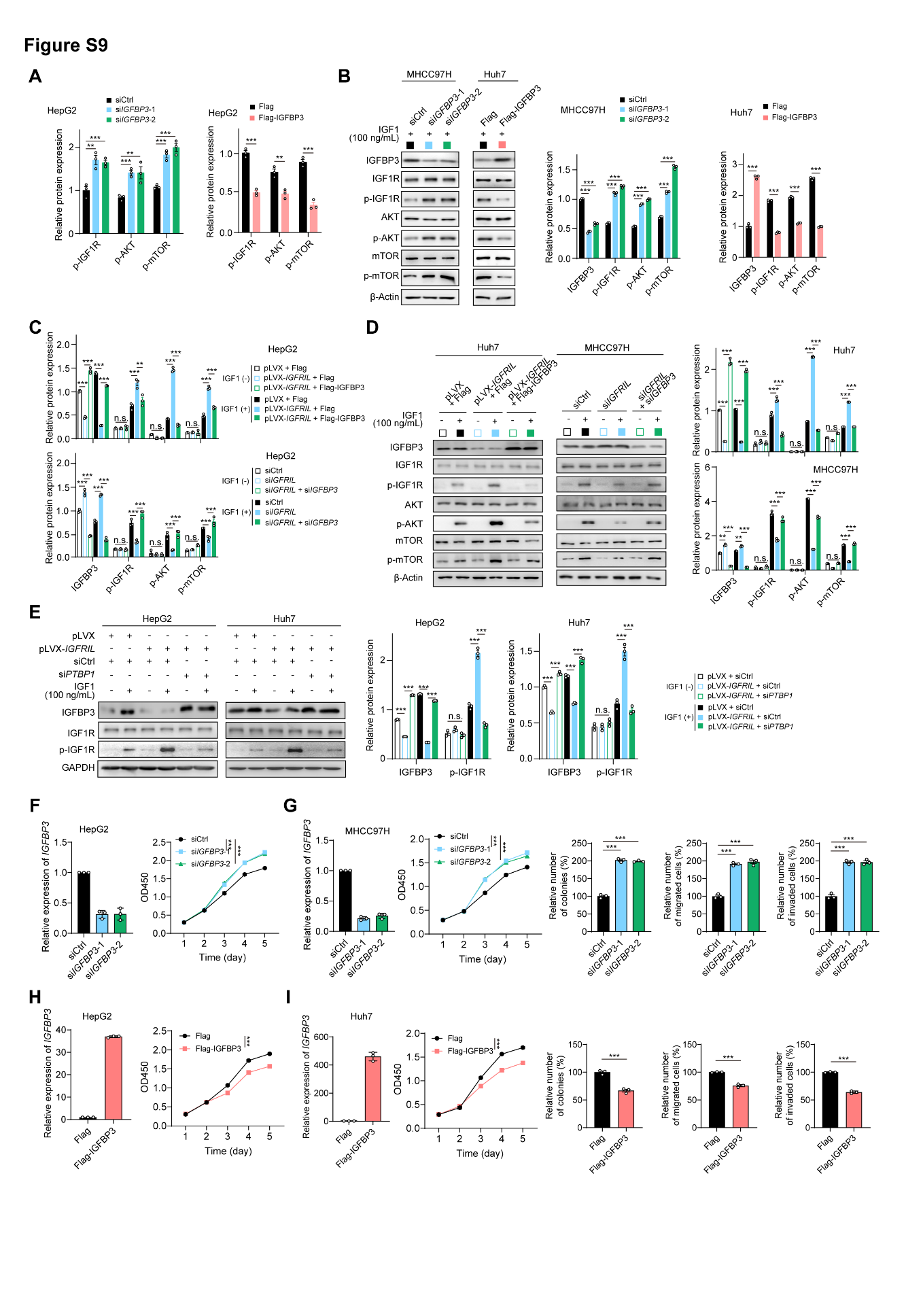


**Figure S9. IGFBP3 reduces the activities of the AKT-mTOR signaling and plays a tumor-suppressive role in HCC cells.**

(A) The quantified immunoblot (IB) band intensities of p-IGF1R, p-AKT and p-mTOR in HepG2 cells upon knockdown (left) or overexpression of IGFBP3 (right) shown in Figure 6A. (B) The effects of *IGFBP3* knockdown in MHCC97H cells (left) or IGFBP3 overexpression in Huh7 cells (right) on activities of the IGF1R-AKT-mTOR axis, upon without or with IGF1 treatment (100 ng/mL). (C) The quantified IB band intensities of IGFBP3, p-IGF1R, p-AKT and p-mTOR in IGFRIL-overexpressed HepG2 cells upon overexpression of IGFBP3 (top), or in *IGFRIL*-depleted HepG2 cells upon knockdown of *IGFBP3* (bottom) shown in Figure 6B. (D) The effects of IGFBP3 overexpression on increased activities of the IGF1R-AKT-mTOR axis by *IGFRIL* overexpression in Huh7 cells (left), or *IGFBP3* knockdown on decreased activities of the IGF1R-AKT-mTOR axis by *IGFRIL* knockdown in MHCC97H cells (right), upon without or with IGF1 treatment (100 ng/mL). (E) The effects of *PTBP1* knockdown on the decreased IGFBP3 levels and increased phosphorylated IGF1R (p-IGF1R) levels by *IGFRIL* overexpression in HepG2 (left) or Huh7 cells (right), upon without or with IGF1 treatment (100 ng/mL). (F) and (G) The effects of *IGFBP3* knockdown on proliferation, plate colony formation, migration and invasion in HepG2 (F) and MHCC97H (G) cells. (H) and (I) The effects of IGFBP3 overexpression on proliferation, plate colony formation, migration and invasion in HepG2 (H) and Huh7 (I) cells. The results are expressed as mean ± standard error of mean (SEM) of three or more independent experiments. Statistical analysis was performed using the 2-sided, 1-way ANOVA (A-G) or unpaired Student’s *t* test (H and I). ^**^, *P* < 0.01; ^***^, *P* < 0.001; n.s., not significant.


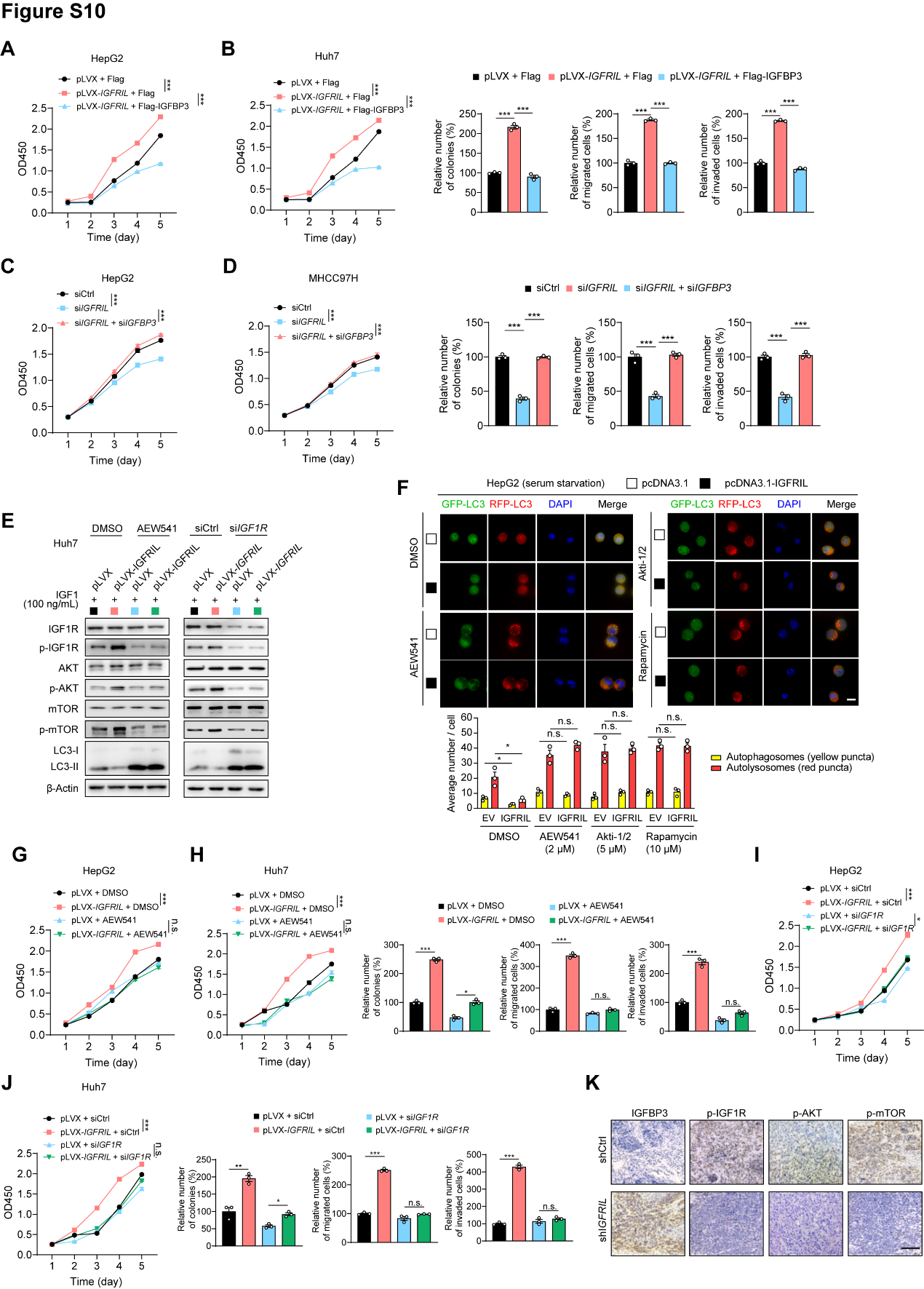


**Figure S10. *IGFRIL* couples with PTBP1 to reduce *IGFBP3* expression to activate the IGF1R-AKT-mTOR signaling and promote HCC progression.**

(A) and (B) The effects of IGFBP3 overexpression on the increased proliferation, plate colony formation, migration and invasion by *IGFRIL* overexpression in HepG2 (A) and Huh7 (B) cells. (C) and (D) The effects of *IGFBP3* knockdown on the decreased proliferation, plate colony formation, migration and invasion by *IGFRIL* knockdown in HepG2 (C) and MHCC97H (D) cells. (E) The effects of IGF1R inhibitor AEW541 (left) or siRNAs against *IGFR* (right) on the increased activities of IGF1R-AKT-mTOR signaling by *IGFRIL* overexpression in Huh7 cells, upon IGF1 treatment (100 ng/mL). (F) *IGFRIL*-mediated suppression of autophagy depends on the IGF1R-AKT-mTOR signaling pathway. The serum-starved HepG2 cells stably expressing mRFP-GFP-LC3 were treated with DMSO, NVP-AEW541 (2 μM), Akti-1/2 (5 μM) or Rapamycin (10 μM) for 12 h, respectively, upon overexpression of *IGFRIL*, followed by staining with DAPI. Red puncta represent the autophagosomes; and yellow puncta in merged picture represent the autolysosomes. Scale bars, 40 µm.

(G) and (H) The effects of IGF1R inhibitor AEW541 on the increased proliferation, plate colony formation, migration and invasion by *IGFRIL* overexpression in HepG2 (G) and Huh7 (H) cells. (I) and (J) The effects of siRNAs against *IGF1R* on the increased proliferation, plate colony formation, migration and invasion by *IGFRIL* overexpression in HepG2 (I) and Huh7 (J) cells. (K) The immunohistochemical (IHC) assays of IGFBP3, p-IGF1R, p-AKT and p-mTOR in subcutaneous tumor tissues from the nude mice transplanted with *IGFRIL*-knocked-down and control HepG2 cells. Scale bar, 200 μm. The results are expressed as mean ± standard error of mean (SEM) of three or more independent experiments. Statistical analysis was performed using the 2-sided, 1-way ANOVA (A-G). ^*^, *P* < 0.05; ^**^, *P* < 0.01; ^***^, *P* < 0.001; n.s., not significant.


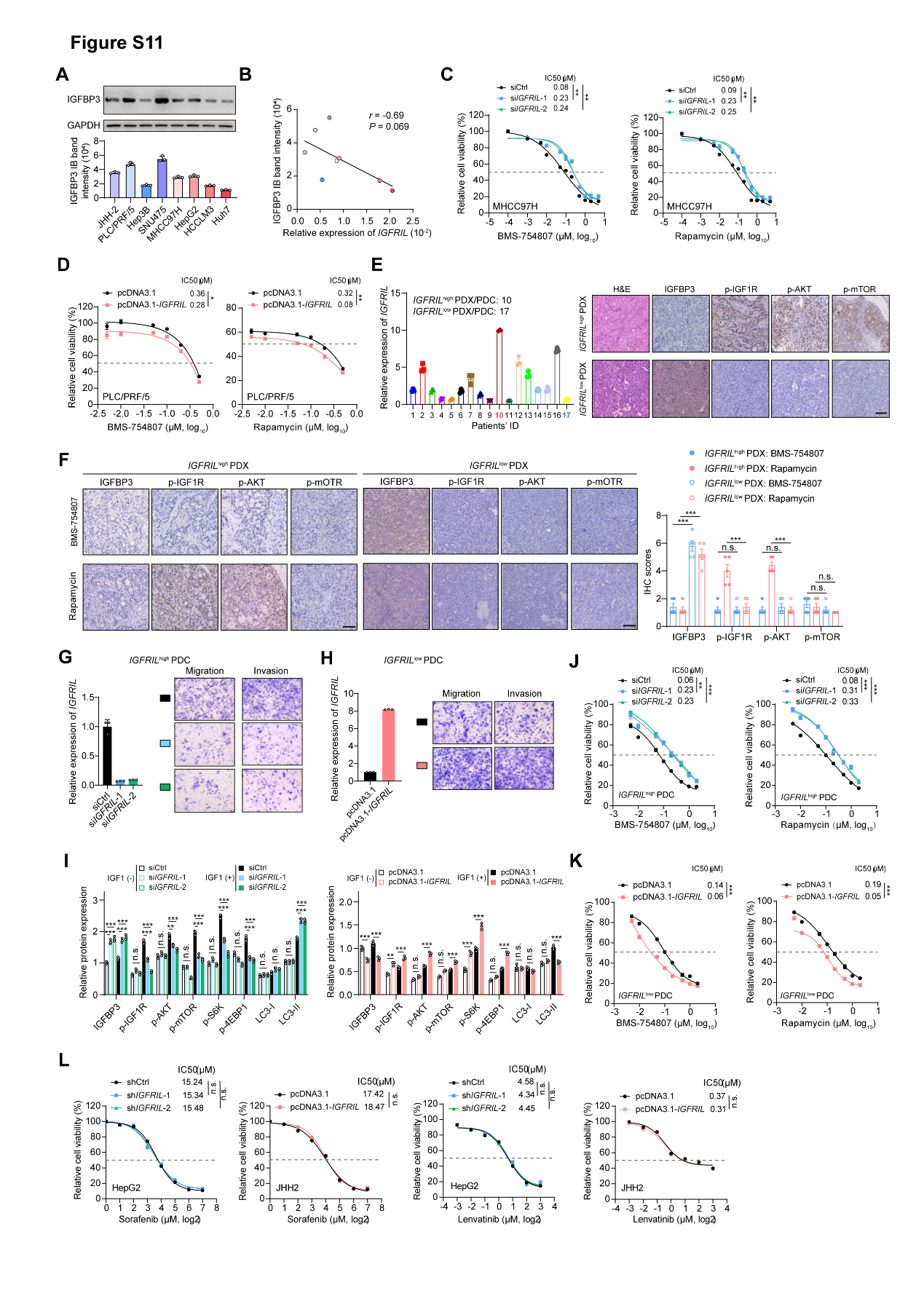


**Figure S11. Upregulation of *IGFRIL* sensitizes HCC cells to IGF1R and mTOR inhibitors.**

(A) The levels of IGFBP3 protein in eight types of HCC cell line determined by immunoblotting assays. (B) The Spearman’s correlation between the levels of *IGFRIL* and IGFBP3 protein in eight types of HCC cell line. (C) The effects of *IGFRIL* knockdown in MHCC97H cells (with higher levels of endogenous *IGFRIL*) on their sensitivities to inhibitors against IGF1R (BMS-754807; left) or mTOR (rapamycin; right). The effects of BMS-754807 or rapamycin on MHCC97H cells growth were determined by CCK-8 assays. IC_50_, half maximal inhibitory concentrations. (D) The effects of *IGFRIL* overexpression in PLC/PRF/5 cells (with lower levels of endogenous *IGFRIL*) on their sensitivities to BMS-754807 (left) or rapamycin (right). (E) Relative expression levels of *IGFRIL* in tumor tissues from a total of 17 HCC PDX models. The PDX models derived from tumor tissues of the patient 10 with the highest *IGFRIL* expression (designated as *IGFRIL*^high^), and patient 17 with lower *IGFRIL* expression (designated as *IGFRIL*^low^) were used for drug sensitivity testing. Right, representative hematoxylin and eosin (H&E) staining, and immunohistochemical (IHC) assays for IGFBP3, p-IGF1R, p-AKT and p-mTOR in tumor tissues from the *IGFRIL*^high^ and *IGFRIL*^low^ PDX models. Scale bar, 200 μm. PDX, patient-derived xenograft; PDC, patient-derived primary tumor cells. (F) The IHC assays for IGFBP3, p-IGF1R, p-AKT and p-mTOR in tumor tissues from the *IGFRIL*^high^ and *IGFRIL*^low^ PDX models that were treated with BMS-754807 or rapamycin. Scale bar, 200 μm. *P* values were calculated using Wilcoxon signed-rank test. (G) and (H) The effects of *IGFRIL* knockdown (G) or overexpression (H) on the cell growth, migration and invasion of the PDCs from the *IGFRIL*^high^ and *IGFRIL*^low^ PDX models. (I) The quantified immunoblot (IB) band intensities of IGFBP3, p-IGF1R, p-AKT, p-mTOR, p-S6K, p-4EBP1, LC3-I and LC3-II in *IGFRIL*^high^ PDCs upon *IGFRIL* knockdown or in *IGFRIL*^low^ PDCs upon *IGFRIL* overexpression shown in Figure 7G. (J) The effects of *IGFRIL* knockdown in *IGFRIL*^high^ PDCs on their sensitivities to BMS-754807 or rapamycin. (K) The effects of *IGFRIL* overexpression in *IGFRIL*^low^ PDCs on their sensitivities to BMS-754807 or rapamycin. (L) The effects of *IGFRIL* knockdown in HepG2 cells or *IGFRIL* overexpression in JHH2 cells on their sensitivities to sorafenib or lenvatinib. The results are expressed as mean ± standard error of mean (SEM) of three or more independent experiments. Statistical analysis was performed using the 2-sided, 1-way ANOVA (C, I, J and L), unpaired Student’s *t* test (D and K) or Wilcoxon signed-rank test (F). ^*^, *P* < 0.05; ^**^, *P* < 0.01; ^***^, *P* < 0.001; n.s., not significant.
